# Identification of a Novel Trifluoromethyl-Bearing Flavonoid as a Promising Androgen Receptor Antagonist: Structure-Based Virtual Screening and In Vitro Study

**DOI:** 10.34133/csbj.0038

**Published:** 2026-04-13

**Authors:** Phakkhathorn Tadasee, Tanatorn Khotavivattana, Kulathida Chaithirayanon, Suvichada Assawakosri, Bodee Nutho

**Affiliations:** ^1^ Department of Pharmacology, Faculty of Science, Mahidol University, Bangkok 10400, Thailand.; ^2^ Center of Excellence in Natural Product, Department of Chemistry, Faculty of Science, Chulalongkorn University, Bangkok 10330, Thailand.; ^3^ Department of Anatomy, Faculty of Science, Mahidol University, Bangkok 10400, Thailand.

## Abstract

Aberrant androgen receptor (AR) expression is the primary driver of prostate cancer progression. While androgen deprivation therapy and AR antagonists are the cornerstones of AR-signaling suppression, their long-term efficacy is frequently compromised by adverse effects and the emergence of drug resistance. Consequently, there is an urgent need to identify novel therapeutic agents. In this study, we screened a library of 112 nonsteroidal compounds (flavonoids and chalcones derivatives) using an integrative in silico and in vitro approach. Initial molecular docking revealed that compounds **18ad**, **18ai**, and **18aj** possessed markedly favorable docking scores than the clinical reference, enzalutamide. Subsequent 300-ns molecular dynamics simulations supported the structural stability of these complexes and identified critical interacting residues, including L704, G708, M742, M745, V746, M749, F764, L873, and T877. Notably, in vitro assays demonstrated that compound **18ad**, a flavone derivative bearing a trifluoromethyl group, substantially suppressed the proliferation of androgen-dependent (LNCaP) cells. Furthermore, **18ad** treatment effectively down-regulated the expression of both the AR and its primary downstream transcriptional target, prostate-specific antigen. Collectively, these findings highlight compound **18ad** as a promising lead scaffold for the development of targeted AR therapies for prostate cancer management.

## Highlights

• Flavonoid **18ad** was identified as a promising androgen receptor antagonist.
• MD simulations and MM/PB(GB)SA suggest the stability of the AR–**18ad** complex.
• **18ad** selectively inhibits AR-dependent LNCaP cells over AR-independent PC-3 cells.
• **18ad** effectively down-regulates the AR/PSA transcriptional signaling axis.

## Graphical Abstract

**Figure:**
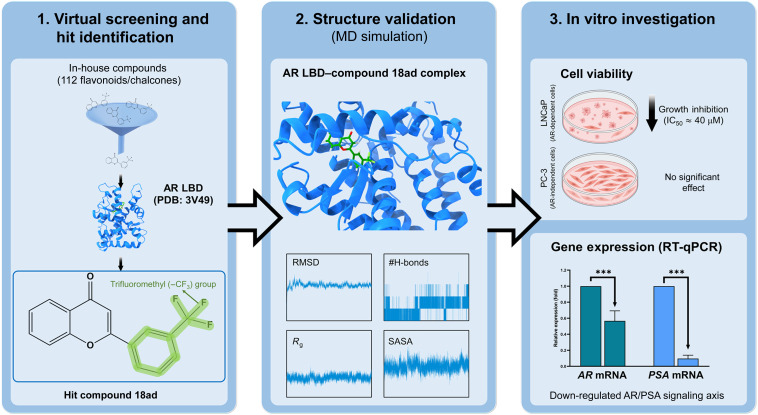

## Introduction

Prostate cancer (PCa) remains the second leading cause of cancer-related mortality among men, with high incidence rates reported across Europe, the United States, and Asia [1]. Current therapeutic standards, including androgen-deprivation therapy and androgen receptor (AR) antagonists, serve as first-line treatments to suppress PCa growth [2]. These agents function by blocking AR activity and nuclear translocation, thereby disrupting androgen-mediated transcriptional programs essential for cancer cell survival [3]. However, the long-term clinical efficacy of these antagonists is frequently compromised by adverse effects—such as edema, hypokalemia, fatigue, and hypertension—and the inevitable emergence of drug resistance. This resistance typically leads to disease progression, increased tumor aggressiveness, and metastasis, characterizing the transition to castration-resistant PCa [4].

Aberrant AR expression is a critical driver of PCa development, particularly when signaling levels are sustained or elevated despite low-androgen environments [5]. Structurally, the AR is composed of 3 primary functional domains: the N-terminal domain, the DNA-binding domain, and the C-terminal ligand-binding domain (LBD) [5,6]. Because the LBD coordinates the binding of both natural androgens and therapeutic antagonists to regulate gene transcription, it remains the primary focal point for the design of next-generation PCa therapeutics [6].

Recently, there has been growing interest in natural products due to their diverse biological activities, such as antioxidant [7], anti-inflammatory [8], and antibacterial effects [9,10]. Moreover, these compounds have demonstrated the ability to modulate various physiological pathways, such as reducing the expression of the estrogen-sensitive gene *CYP7a1*, regulating the follicle-stimulating hormone/luteinizing hormone ratio [11], and decreasing de novo lipogenesis in several cancer types [12,13]. Specifically, nonsteroidal scaffolds, especially flavonoids and chalcones, have shown marked therapeutic potential against PCa. Chalcones are known to inhibit cell proliferation by inducing p53 expression to drive cell cycle arrest and stimulating caspase-dependent apoptosis [14]. Similarly, flavonoids can suppress PCa growth and migration by inhibiting monoamine oxidase isoenzymes and targeting epigenetic regulators such as DNA methyltransferases and histone deacetylases [15,16]. Despite their potential, the clinical application of natural compounds is often hindered by the high dosages required to achieve efficacy, which can lead to off-target toxicity or further resistance [17]. Consequently, research has shifted toward the design of semisynthetic derivatives aimed at enhancing potency and suppressing AR expression at lower effective concentrations, thereby minimizing adverse effects [18,19].

This study aimed to screen an in-house library of 112 semisynthetic flavonoids and chalcones to investigate their potential as AR LBD antagonists using an integrative computational and experimental pipeline. Initial screening employed molecular docking to determine binding modes and interactions toward the AR LBD, along with SwissADME analysis to predict physicochemical properties and drug-likeness. The structural stability and binding free energies of the resulting AR LBD–ligand complexes were further assessed by molecular dynamics (MD) simulations. Finally, the therapeutic potential of the hit compound to modulate the AR/prostate-specific antigen (PSA) axis was validated in vitro using both androgen-dependent (LNCaP) and androgen-independent (PC-3) PCa cell lines.

## Materials and Methods

### Computational studies

#### System preparation and molecular docking

The x-ray crystal structure of the AR LBD (Protein Data Bank [PDB] ID: 3V49 [20]) was obtained from the RCSB PDB (http://www.rcsb.org/). This structure was selected because it is cocrystallized with a nonsteroidal selective AR modulator that is similar to the nonsteroidal scaffolds of flavonoids and chalcones, thereby providing a relevant conformation for identifying novel nonsteroidal antagonists. Moreover, to date, a definitive complex structure of the wild-type AR LBD bound to a pure antagonist has not been elucidated, which presents a significant challenge in modeling antagonistic modulation. Therefore, the agonistic conformation (3V49) was used for virtual screening to examine protein–ligand binding, following protocols used in previous studies [21–23]. To prepare the protein for molecular docking, all solvent molecules and cocrystallized ligand were removed. The SMILES (Simplified Molecular Input Line Entry System) representation of enzalutamide was obtained from the PubChem database (PubChem CID: 15951529, http://pubchem.ncbi.nlm.nih.gov/), while the structures of the 112 flavonoids (89 compounds) and chalcones (23 compounds) were sourced from previous studies [24,25] (Tables S1 to S3). These compounds were converted into 3-dimensional (3D) PDB format using the Online SMILES Translator and Structure File Generator (http://cactus.nci.nih.gov/translate/). PDBQT files for both the AR protein and the compounds were generated using AutoDockFR 1.0 [26], and molecular docking was subsequently performed with AutoDock Vina 1.2.5 [27]. The docking grid center was defined based on the coordinates of the cocrystallized ligand within the active site. A grid box of 20 × 20 × 20 Å (centered at *x* = 27.30, *y* = 2.07, *z* = 4.62) was selected to fully encompass the ligand-binding pocket. The exhaustiveness parameter was set to 64 to ensure adequate sampling of conformational space. To validate the reliability of this docking protocol, a redocking simulation was performed. Superimposition of the best-scored docked pose with the cocrystallized ligand (PK0) indicated good alignment with the native crystal conformation, yielding a root-mean-square deviation (RMSD) of 1.12 Å (Fig. S1). Furthermore, protocol performance was evaluated using a small validation set of known AR agonists and antagonists to verify the workflow’s ranking and discrimination capabilities. The docking results revealed that established AR antagonists generally clustered within more favorable energy ranges, exhibiting more negative docking scores on average compared to AR agonists (Table S4). While molecular docking scoring functions are inherently approximate, this preferential scoring trend for antagonists shows that the chosen docking parameters are dependable for subsequent virtual screening and capable of effectively enriching novel AR antagonists. Following screening, the docked conformations of the candidate compounds exhibiting the lowest binding energies were selected as starting structures for MD simulations. Protein–ligand interactions were analyzed and visualized using ChimeraX [28] for 3D structural evaluation and Discovery Studio Visualizer (BIOVIA, San Diego, CA, USA) for 2-dimensional interaction diagrams.

#### Physicochemical properties and drug-likeness predictions

Physicochemical properties and drug-likeness were predicted using the SwissADME web server [29]. The SwissADME platform incorporates several predictive models and visualization tools, including the Bioavailability Radar, iLOGP, and the BOILED-Egg model. The BOILED-Egg model provides a robust graphical representation to predict gastrointestinal absorption and blood–brain barrier (BBB) permeation potential. This model distinguishes between well-absorbed and poorly absorbed molecules by plotting their topological polar surface area against their lipophilicity (WLOGP), based on an adaptation of Egan’s egg model [30]. Additionally, the iLOGP method provides an accurate estimation of lipophilicity by employing a physics-based fragmental method [31].

#### MD simulations

MD simulations of the AR LBD–ligand complexes were performed using the AMBER 24 software package [32,33], utilizing the SANDER (Simulated Annealing with NMR-Derived Energy Restraints) and PMEMD (Particle Mesh Ewald Molecular Dynamics) modules. The simulation protocol followed our previously established methodology [34,35]. Briefly, atomic charges for enzalutamide and the candidate compounds (**18ad**, **18ai**, and **18aj**) were assigned using the Antechamber module [36], and the ligands were parameterized using the General AMBER Force Field 2 (GAFF2) [37]. The ff19SB force field [38] was applied to the protein. The system was solvated using the optimal point charge explicit water model [39], ensuring a minimum solute–wall distance of 10 Å. Chloride (Cl^−^) counterions were added to neutralize the overall system charge. Following standard procedures, the system underwent energy minimization, heating, and density equilibration. Subsequently, the production phase was carried out under an isothermal–isobaric (NPT) ensemble at 310 K and 1 atm until a total simulation time of 300 ns. Simulations were performed in duplicate for each system, yielding 2 independent 300-ns replicates with different initial velocities. The postsimulation trajectories were analyzed for structural features using the CPPTRAJ (C++ Process TRAJectory) module [40], while the total binding free energies and per-residue decomposition energies were calculated using the MMPBSA.py module [41].

### In vitro studies

#### Materials

Compound **18ad** was synthesized and purified in-house as previously described [25]. Enzalutamide was purchased from MedChemExpress (Monmouth Junction, NJ, USA). Stock solutions of enzalutamide and compound **18ad** were prepared in 100% dimethyl sulfoxide (DMSO; Sigma-Aldrich, St. Louis, MO, USA) at a concentration of 50 mM and stored at 4 °C until use. Prior to each experiment, stock solutions were freshly diluted in culture medium to obtain the desired working concentrations. The final DMSO concentration in the culture medium was maintained below 0.2% (v/v) to minimize solvent-related cytotoxic effects.

#### Cell cultures and reagents

Human PCa cell lines, LNCaP (ATCC CRL-1740) and PC-3 (ATCC CRL-1435), were purchased from the American Type Culture Collection (ATCC, Manassas, VA, USA). Cells were cultured and maintained in RPMI 1640 medium (Thermo Fisher Scientific/Gibco, Grand Island, NY, USA) supplemented with 10% fetal bovine serum (Thermo Fisher Scientific/Gibco), 100 U/ml penicillin, and 100 μg/ml streptomycin. Cultures were incubated at 37 °C in a humidified incubator containing 5% CO_2_.

#### Cell viability assay

PCa cells (LNCaP and PC-3) were seeded in 96-well plates at a density of approximately 1 × 10^4^ cells per well in triplicate and incubated overnight. Subsequently, cells were treated with various concentrations (3.125, 6.25, 12.5, 25, 50, and 100 μM) of the compound or a vehicle control (0.2% DMSO) for 24 and 48 h. Cell viability was then assessed using the MTS (3-(4,5-dimethylthiazol-2-yl)-5-(3-carboxymethoxyphenyl)-2-(4-sulfophenyl)-2H-tetrazolium) assay (Promega, Madison, WI, USA) in accordance with the manufacturer’s instructions.

#### Reverse transcription-quantitative polymerase chain reaction

Total RNA was extracted from LNCaP cells treated with enzalutamide, compound **18ad** or the vehicle control using TRIzol reagent (Invitrogen, Carlsbad, CA, USA). Subsequently, cDNA was synthesized using an iScript cDNA Synthesis Kit (Bio-Rad, Hercules, CA, USA). The quantitative polymerase chain reaction (qPCR) samples were prepared using iQ SYBR Green Supermix (Bio-Rad) with specific primers for *AR*, *PSA*, and *β-actin* [12]. The qPCR analysis was performed using the CFX Connect Real-Time System (Bio-Rad). Data are presented as the mean ratio of triplicates normalized to the internal control, *β-actin*.

#### Statistical analysis

IBM SPSS Statistics 31.0.1.0 and GraphPad Prism 9.0 software were used for all statistical analyses and the generation of graphs. To assess cell viability, a 1-way analysis of variance (ANOVA), followed by the Games–Howell post hoc test, was employed to compare all possible pairwise group combinations and mean differences among experimental groups. Gene expression levels were normalized to *β-actin* and analyzed using the ΔCt method. Differences between treatment and control groups were evaluated using an unpaired *t* test and 2-way ANOVA, as appropriate. Data are presented as the means ± standard error of the mean (SEM) derived from at least 3 independent experiments. Statistical significance was defined as *P* < 0.05 (*P < 0.05, **P < 0.01, and ***P < 0.001).

## Results and Discussion

### Structure-based virtual screening

**Fig. 1.**
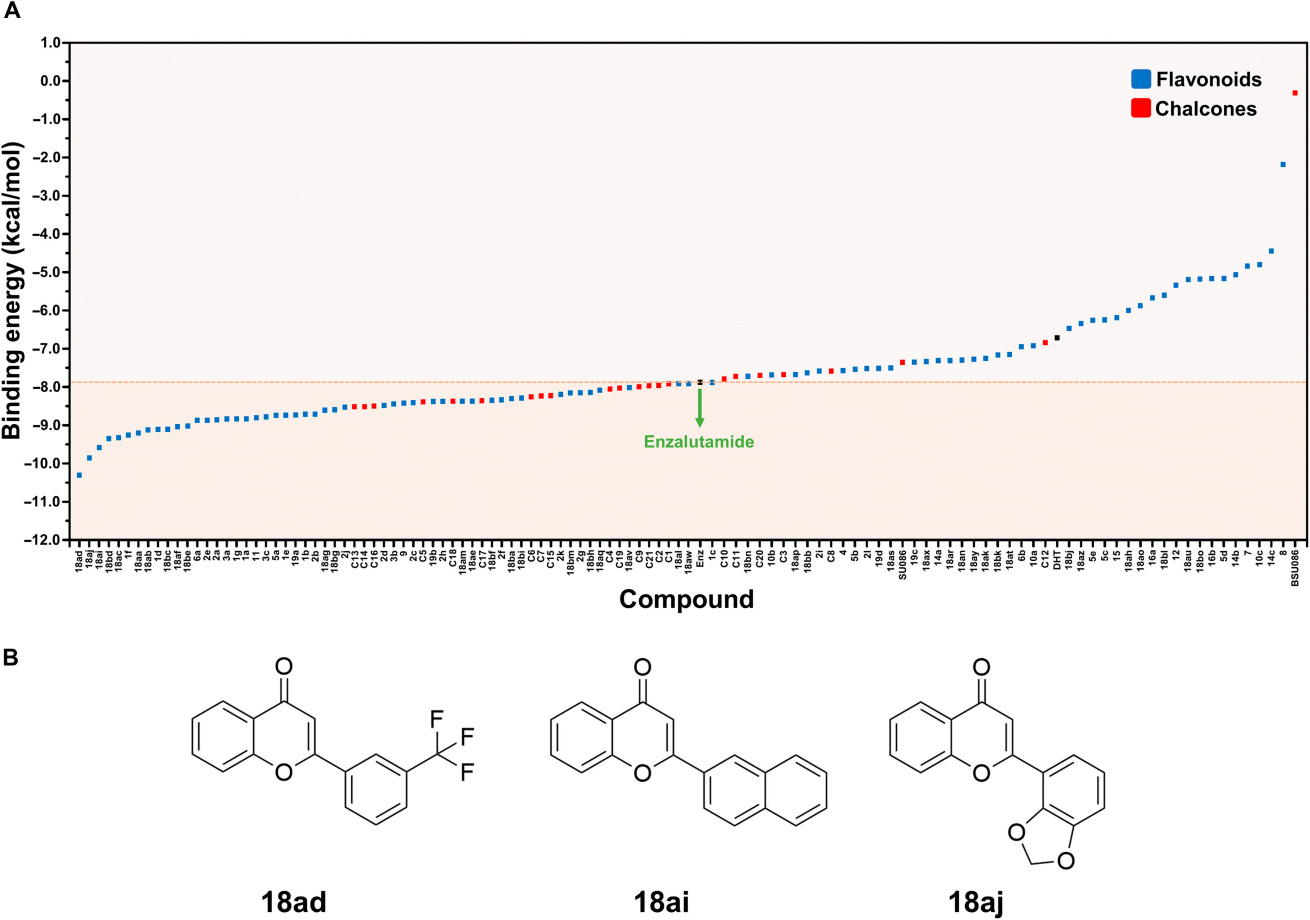
(A) Ranking of AutoDock Vina docking scores for the 112 in-house compounds, ordered from lowest (most favorable) to highest binding energy. (B) The top 3 candidate compounds (18ad, 18ai, and 18aj) selected for further analysis based on their highly favorable docking scores.

We performed molecular docking to evaluate the binding modes and docking scores of 112 in-house compounds against the AR LBD. Since more negative docking scores indicate stronger predicted interactions within the binding pocket, our screening identified 63 compounds with favorable binding profiles. Among these, flavonoids **18ad**, **18ai**, and **18aj** exhibited the most favorable docking scores (−10.30, −9.58, and −9.85 kcal/mol, respectively). These values were more negative than those of the reference drug enzalutamide (−7.87 kcal/mol), as shown in Fig. 1A. However, it is important to note that docking scores represent a relative ranking metric rather than a quantitative measure of binding affinity. Docking scoring functions employ simplified approximations of solvation effects, protein flexibility, and entropic contributions, which can lead to discrepancies when compared with more physics-based free energy approaches [42,43]. Accordingly, the docking results presented here were interpreted primarily as an initial screening filter, rather than definitive indicators of superior binding strength compared to the reference drug. To rigorously evaluate the binding stability of the top candidates, we subsequently conducted MD simulations and free energy analyses (see the “Binding free energy calculations and key binding residues” section).

**Fig. 2.**
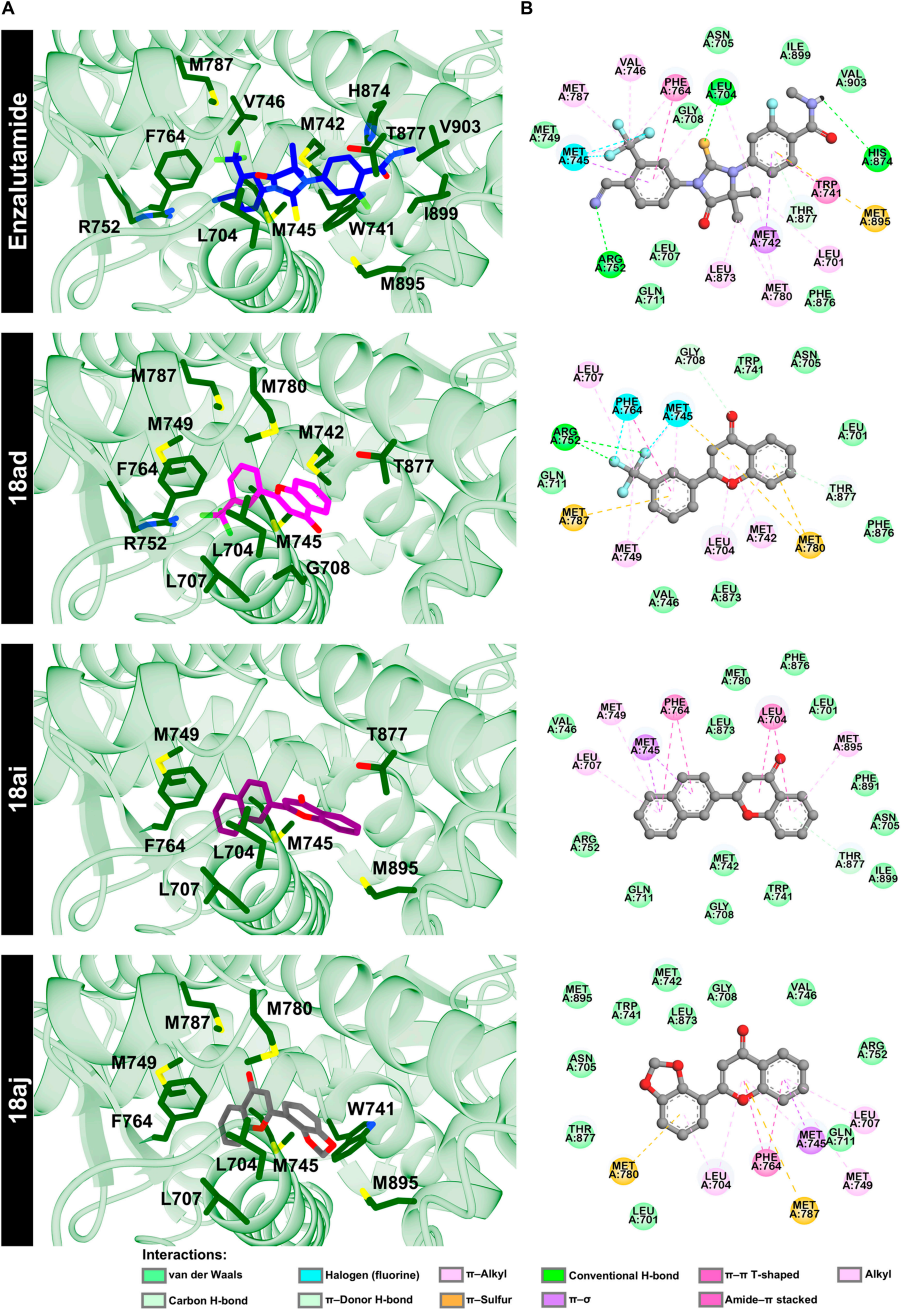
Structural analysis of enzalutamide and the top 3 compounds. (A) Three-dimensional (3D) structural representations and (B) 2-dimensional (2D) interaction diagrams of enzalutamide and compound 18ad, 18ai, and 18aj complexed with the androgen receptor (AR) ligand-binding domain (LBD), as determined by molecular docking.

Despite differences in peripheral substituents (ring B) of compounds **18ad**, **18ai**, and **18aj** (Fig. 1B), these molecules share a common flavone-based scaffold characterized by a planar aromatic framework, which likely facilitates hydrophobic and π interactions within the AR ligand-binding pocket. Variations in substituent patterns, including the trifluoromethyl (CF_3_), naphthalene, and benzodioxole moieties in compounds **18ad**, **18ai**, and **18aj**, respectively, appear to influence ligand orientation and interaction patterns, suggesting potential structure–interaction relationships among the top-ranked compounds. To gain further structural insight, the 3D structures and 2-dimensional interaction diagrams of **18ad**, **18ai**, and **18aj** in complex with the AR LBD were analyzed (Fig. 2). The binding poses of these compounds within the active site were identified and compared with those of enzalutamide. The top 3 candidate compounds exhibited interactions with key residues in the AR LBD, with binding conformations comparable to those of the reference drug. These unique interactions involved several key residues, as shown in Fig. 2B and Table S5.

Enzalutamide, a standard AR antagonist, served as the benchmark for these comparisons. We found that its specific substituents, particularly –CF_3_ and cyano (–CN) groups, engage in a halogen bond with M745 and a hydrogen bond with R752, respectively. Previous reports have established that critical contact patterns for enzalutamide involve interactions with residues M745, M749, R752, and F764, which are located in close proximity to the coactivator interaction site within the AR LBD [44]. Our findings indicate that the variations in substituent groups among the hit compounds influenced protein–ligand binding interactions. For instance, the 1,3-benzodioxole moiety of **18aj** contacted L704 and M780 via π–alkyl and π–sulfur interactions, respectively. In contrast, the naphthalene ring of **18ai** interacted with L707 and M749 through π–alkyl interactions, while also engaging M745 and F764 via π–sigma and amide–π stacked interactions. Notably, the phenyl ring of **18ad**, which incorporates an *m*-trifluoromethyl group, interacted with M745, R752, and F764 with interaction types comparable to those observed for enzalutamide. Specifically, the trifluoromethyl moiety formed a hydrogen bond with R752 and halogen bonds with M745 and F764. Likewise, a previous study revealed that substitution at the 4′ position of flavones with a fluorine group leads to strong binding to the AR LBD through interactions with residue R752, acting as a potent antagonist [45]. These findings are consistent with our results for compound **18ad**, which involves these critical residues and may contribute to stabilization of the protein–ligand complex. However, docking-derived interaction patterns alone do not establish definitive structure–activity relationships, and further experimental validation is required.

### Physicochemical properties and drug-likeness predictions

**Fig. 3.**
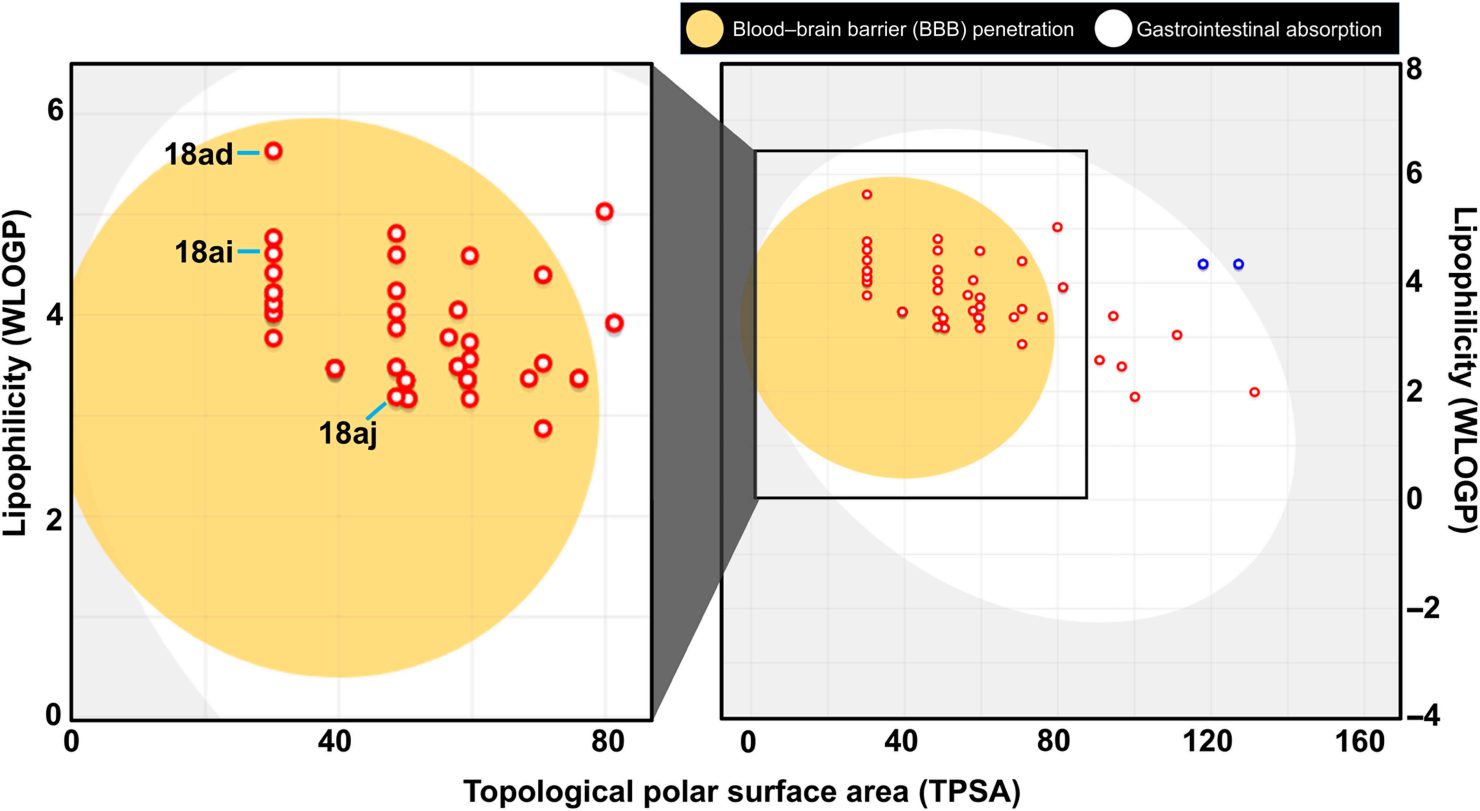
BOILED-Egg plot evaluation of the 63 compounds with more negative predicted docking scores than enzalutamide. The diagram illustrates the predicted gastrointestinal absorption and brain penetrability for the selected compounds, highlighting the positions of hit compounds 18ad, 18ai, and 18aj.

The physicochemical properties and drug-likeness of the 63 compounds identified as having docking scores superior or comparable to enzalutamide (indicated by the orange region in Fig. 1A) were evaluated using SwissADME. This platform incorporates Lipinski’s “Rule of Five” [46] to assess oral bioavailability, along with several predictive lipophilicity models, including MLOGP [47], WLOGP [31], SILICOS-IT [48], and iLOGP [49], as provided in Table S6. Passive gastrointestinal absorption and BBB permeability were predicted using the BOILED-Egg model, which utilizes the physicochemical descriptors WLOGP and topological polar surface area to map lipophilicity and polarity [29,50]. The results indicated that most compounds possess favorable bioavailability, with a majority falling within the regions of high gastrointestinal absorption and BBB permeability (the yolk region) on the BOILED-Egg plot (Fig. 3). It is known that certain AR antagonists, including enzalutamide, are associated with central nervous system-related adverse effects, such as seizures, which may arise from significant brain exposure and off-target interactions with the gamma-aminobutyric acid-gated chloride channel [51,52]. Therefore, the predicted BBB permeability of the identified compounds was considered from a safety perspective. The BOILED-Egg analysis suggests that several compounds occupy regions associated with passive BBB permeation, indicating physicochemical properties consistent with efficient membrane transport. However, this model evaluates only passive permeability and does not account for active transport processes or specific off-target interactions. Therefore, these in silico predictions should be interpreted cautiously and do not directly establish neurological safety or seizure risk. Future investigations such as receptor profiling and in vivo neurotoxicity models are required to comprehensively assess central nervous system liabilities during lead optimization.

In addition, the molecular weights and number of hydrogen bond acceptors for all 63 compounds remained within the recognized thresholds of <500 Da and <10, respectively, adhering strictly to Lipinski’s criteria with zero violations (Table S6). To provide a comparative benchmark, we also evaluated the physicochemical profile of the reference drug enzalutamide. According to the BOILED-Egg analysis, optimal lipophilicity (LogP) values typically range between 0 and 3. While compound **18aj** fell within this optimal range (LogP = 3.11), compounds **18ad** and **18ai** exhibited consensus LogP values of 4.21 and 4.09, respectively. Although a LogP > 4 approaches the upper boundary of optimal oral drug space, such relatively high lipophilicity and moderate-to-poor water solubility are typical inherent limitations of natural flavonoid and chalcone scaffolds [53,54]. Consequently, these parameters represent primary targets for future hit-to-lead optimization; furthermore, their aqueous solubility and oral bioavailability can be successfully addressed downstream utilizing advanced drug formulation and delivery systems [55,56]. To strengthen the developability assessment, we performed an expanded in silico evaluation. The candidate compounds (**18ad**, **18ai**, and **18aj**) displayed favorable synthetic accessibility scores (<4) and showed no problematic structural alerts based on the Pan Assay Interference Compounds (PAINS) filter. Overall, these findings suggest that the candidate compounds possess favorable drug-likeness and synthetically tractable physicochemical profiles, making them viable lead scaffolds for further development.

### MD simulation

**Fig. 4.**
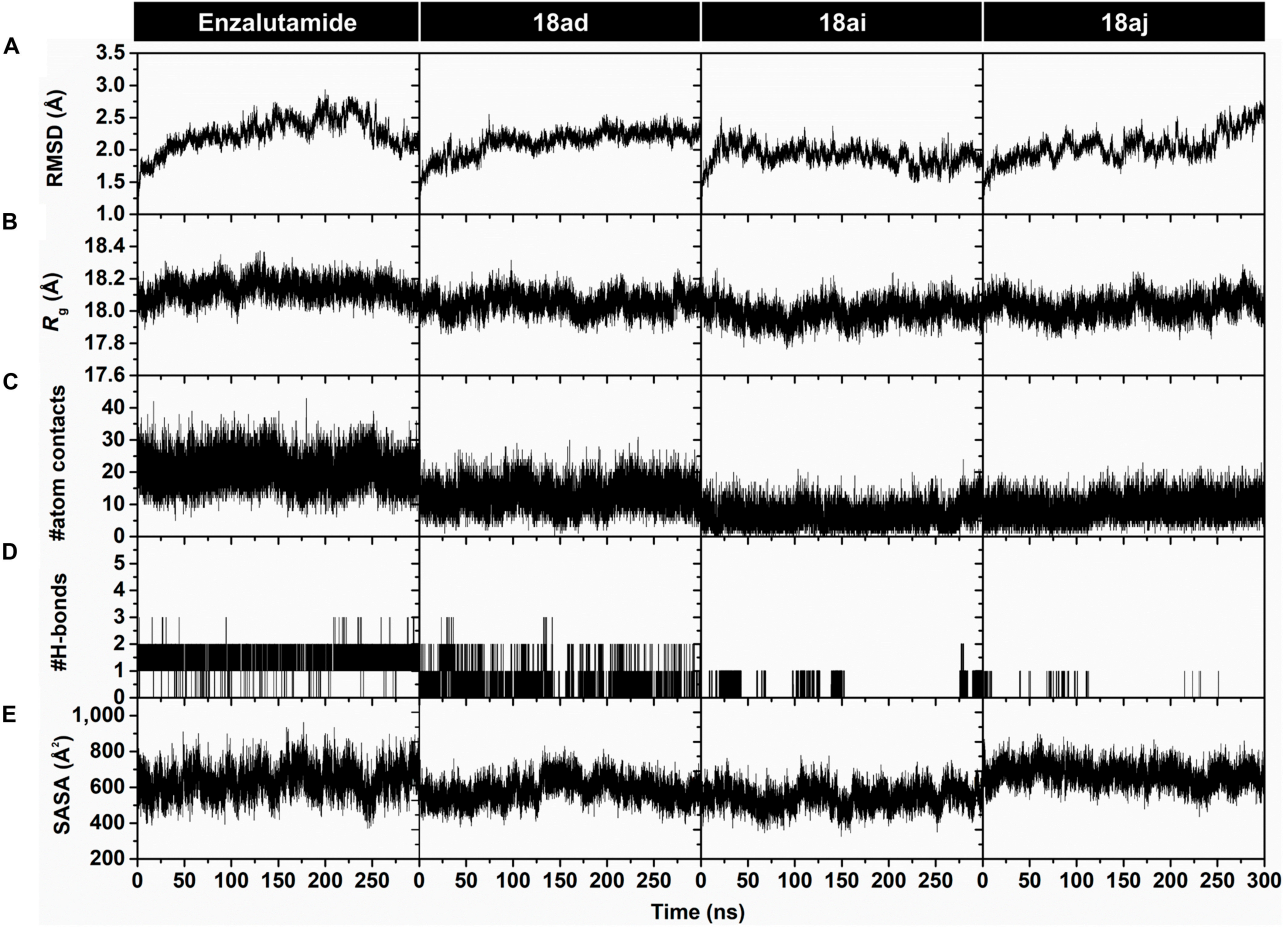
Structural stability and interaction profiles of the androgen receptor (AR) ligand-binding domain (LBD)–ligand complexes during 300 ns of molecular dynamics (MD) simulations (rep #1). Time-dependent plots illustrate (A) root-mean-square deviation (RMSD), (B) radius of gyration (*R*_g_), (C) the number of atomic contacts (#atom contacts), (D) the number of intermolecular hydrogen bonds (#H-bonds), and (E) solvent-accessible surface area (SASA) for enzalutamide and compounds 18ad, 18ai, and 18aj in complex with the AR LBD.

To investigate the structural stability and dynamic behavior of the AR LBD–ligand complexes in an aqueous environment, we performed 300-ns MD simulations in duplicate (2 independent replicates per system, designated as rep #1 and rep #2). The structural stability and convergence of the complexes were evaluated using 5 key parameters across both replicates: RMSD, radius of gyration (*R*_g_), the number of atom contacts (#atom contacts), the number of hydrogen bonds (#H-bonds) between the protein and ligand, and solvent-accessible surface area (SASA) (Fig. 4). Because the 2 independent trajectories exhibited highly consistent convergence profiles, the discussion below focuses on rep #1 as a representative trajectory, while replicate analyses are provided in Fig. S2.

The RMSD profiles of complex atoms indicated that the 3 focused systems were likely to reach a stable conformational regime earlier than the enzalutamide system. These complexes exhibited relatively low RMSD values (below 2.5 Å) and maintained stable fluctuations between ~2.00 and 2.25 Å after approximately 75 ns of simulation (Fig. 4A), consistent with sustained ligand retention within the AR LBD binding pocket. Although compound **18aj** displayed minor fluctuations after 250 ns, these variations remained within a stable range and likely reflect local adjustments in protein–ligand interactions rather than global destabilization.

The *R*_g_ values were analyzed to assess protein compactness upon ligand binding (Fig. 4B). Elevated *R*_g_ values or large variations typically indicate major conformational rearrangements, such as partial unfolding, which may be influenced by binding-site accessibility and protein flexibility [57]. Analysis of the protein C_α_ atoms showed that all complexes remained structurally stable throughout the 300-ns simulations. The *R*_g_ values for compounds **18ad**, **18ai**, and **18aj** fluctuated around 18.05, 18.00, and 18.20 Å, respectively. Similarly, enzalutamide exhibited an average *R*_g_ of 18.15 Å, with modest fluctuations during the first 100 ns prior to stabilization.

To further evaluate binding stability and system convergence, we quantified protein–ligand atom contacts throughout the simulations using a 3.5-Å distance cutoff between each compound and the predefined active site residues (Fig. 4C). The resulting contact profiles exhibited persistent plateau behavior for the 3 complex systems, indicating sustained ligand accommodation within the binding pocket over the course of the simulation. The average #atom contacts calculated over the final 50 ns were 19 ± 5, 13 ± 4, 8 ± 4, and 10 ± 3 for the enzalutamide, **18ad**, **18ai**, and **18aj** systems, respectively. Among the 3 compounds, **18ad** exhibited the highest #atom contacts, suggesting that it may interact more robustly and bind more tightly to the active site residues of the AR LBD compared to the other derivatives.

The #H-bond analysis was evaluated to provide additional insight into protein–ligand interactions during the MD simulations (Fig. 4D). Compound **18ad** exhibited an average of 1 ± 1 H-bonds throughout the simulation, compared with 2 ± 1 for enzalutamide, whereas compounds **18ai** and **18aj** displayed minimal or transient H-bond interactions. Despite these differences, the relatively stable RMSD and #atom contacts profiles observed for **18ai** and **18aj** suggest that alternative noncovalent interactions (e.g., van der Waals and π interactions) contribute to maintaining ligand accommodation within the binding pocket. Furthermore, because complex stability is multifactorial, the H-bonding pattern of **18ad**, which remained comparable to that of enzalutamide, supports a similar interaction mode without implying enhanced stability based solely on bond counts.

The SASA analysis was performed to evaluate solvent exposure of the AR LBD residues located within a 5-Å radius of the ligand-binding pocket. To ensure direct comparability across simulations, we used a predefined set of residues within this 5-Å region (residues L701, L704, N705, E706, L707, G708, R710, S740, W741, L744, M745, F747, A748, W751, L762, Y763, F764, R779, R786, T877, L880, L881, S884, M895, I899, and V903) consistently for all systems. As shown in Fig. 4E, the candidate compounds exhibited generally lower SASA values than enzalutamide (~650 Å^2^), consistent with reduced solvent exposure of the binding interface and reduced solvent accessibility of nearby residues. Compound **18ad** maintained a relatively low average SASA of ~450 Å^2^, although transient increases between 125 and 200 ns indicate brief partial solvent exposure. Compound **18ai** displayed comparable average values with minor early-phase oscillations, whereas compound **18aj** remained relatively stable at ~600 Å^2^ throughout the simulation. Altogether, these structural analyses showed that the simulation models achieved relative stability; therefore, the structural coordinates from the final 50 ns of the simulations were adopted as the production period for further analysis.

### Binding free energy calculations and key binding residues

The binding efficiency of compounds **18ad**, **18ai**, and **18aj** to the AR LBD was estimated using the molecular mechanics/Poisson–Boltzmann surface area and molecular mechanics/generalized Born surface area methods [58] based on 500 snapshots extracted from the final 50 ns of the MD simulations. The total binding free energy (∆*G*_bind_) and its individual energetic components are summarized in Table 1. According to the molecular mechanics (∆*E*_MM_) terms in the gas phase, van der Waals interactions (∆*E*_vdW_) were identified as the primary driving forces for the formation of the protein–ligand complexes across all compounds. These interactions were stronger than the electrostatic interactions (∆*E*_ele_). However, both the ∆*E*_vdW_ and ∆*E*_ele_ contributions for these candidate compounds were numerically weaker than those calculated for enzalutamide. When accounting for the solvation energy (∆*G*_sol_), the ∆*G*_bind_ of compound **18ad**, **18ai**, and **18aj** toward the AR LBD remained higher (less negative) than that of enzalutamide. This apparent discrepancy between the docking scores (“Structure-based virtual screening” section) and the MM/PB(GB)SA free energy estimates reflects the fundamental methodological differences between these computational approaches. Whereas docking relies on static scoring functions that often underestimate solvation penalties and neglect conformational dynamics, MM/PB(GB)SA analyses incorporate ensemble averaging derived from MD simulations and a more rigorous treatment of solvent effects. Consequently, although the candidate compounds did not energetically surpass enzalutamide in these end-state calculations, they maintained consistently negative binding free energies, supporting stable accommodation within the AR ligand-binding pocket. Importantly, these findings position these compounds as structurally promising lead scaffolds rather than higher-affinity replacements for the reference drug, highlighting opportunities for future optimization to improve binding efficiency.

**Table 1.** Predicted binding free energies and their components for the AR LBD–ligand complexes. Values were calculated using MM/PBSA and MM/GBSA methods. Data are presented as means ± SEM.

Energy components	Enzalutamide	18ad	18ai	18aj
**Gas term**				
$∆E_{\mathrm{vdW}}$	–61.90 ± 0.12	–42.01 ± 0.09	–43.04 ± 0.11	–40.59 ± 0.10
$∆E_{\mathrm{ele}}$	–35.64 ± 0.14	0.19 ± 0.09	–8.02 ± 0.08	–7.04 ± 0.09
$∆E_{\mathrm{MM}}$	–97.54 ± 0.16	–41.82 ± 0.12	–51.06 ± 0.12	–47.63 ± 0.12
**Solvation term**				
PBSA				
$∆G_{\mathrm{sol}\left(\text{PBSA}\right)}^{\mathrm{ele}}$	58.54 ± 0.14	23.44 ± 0.13	27.00 ± 0.10	29.19 ± 0.10
$∆G_{\mathrm{sol}\left(\text{PBSA}\right)}^{\text{nonpolar}}$	–4.81 ± 0.01	–3.34 ± 0.01	–3.52 ± 0.01	–3.27 ± 0.01
$∆G_{\mathrm{sol}\left(\text{PBSA}\right)}$	53.74 ± 0.11	20.10 ± 0.13	23.48 ± 0.10	25.93 ± 0.10
GBSA				
$∆G_{\mathrm{sol}\left(\text{GBSA}\right)}^{\mathrm{ele}}$	51.27 ± 0.11	12.06 ± 0.08	19.56 ± 0.08	20.62 ± 0.06
$∆G_{\mathrm{sol}\left(\text{GBSA}\right)}^{\text{nonpolar}}$	–8.27 ± 0.01	–5.71 ± 0.01	–5.53 ± 0.01	–5.19 ± 0.01
$∆G_{\mathrm{sol}\left(\text{GBSA}\right)}$	43.00 ± 0.11	6.35 ± 0.08	14.03 ± 0.08	15.43 ± 0.06
**Binding free energy**				
$∆G_{\text{total}\left(\mathrm{MM}/\text{PBSA}\right)}$	–43.80 ± 0.14	–21.72 ± 0.15	–27.58 ± 0.14	–21.70 ± 0.15
$∆G_{\text{total}\left(\mathrm{MM}/\text{GBSA}\right)}$	–54.54 ± 0.12	–35.47 ± 0.09	–37.03 ± 0.12	–32.20 ± 0.10

AR, androgen receptor; LBD, ligand-binding domain; SEM, standard error of the mean; PBSA, Poisson–Boltzmann surface area; GBSA, generalized Born surface area; MM/PBSA, molecular mechanics/Poisson–Boltzmann surface area; MM/GBSA, molecular mechanics/generalized Born surface area

**Fig. 5.**
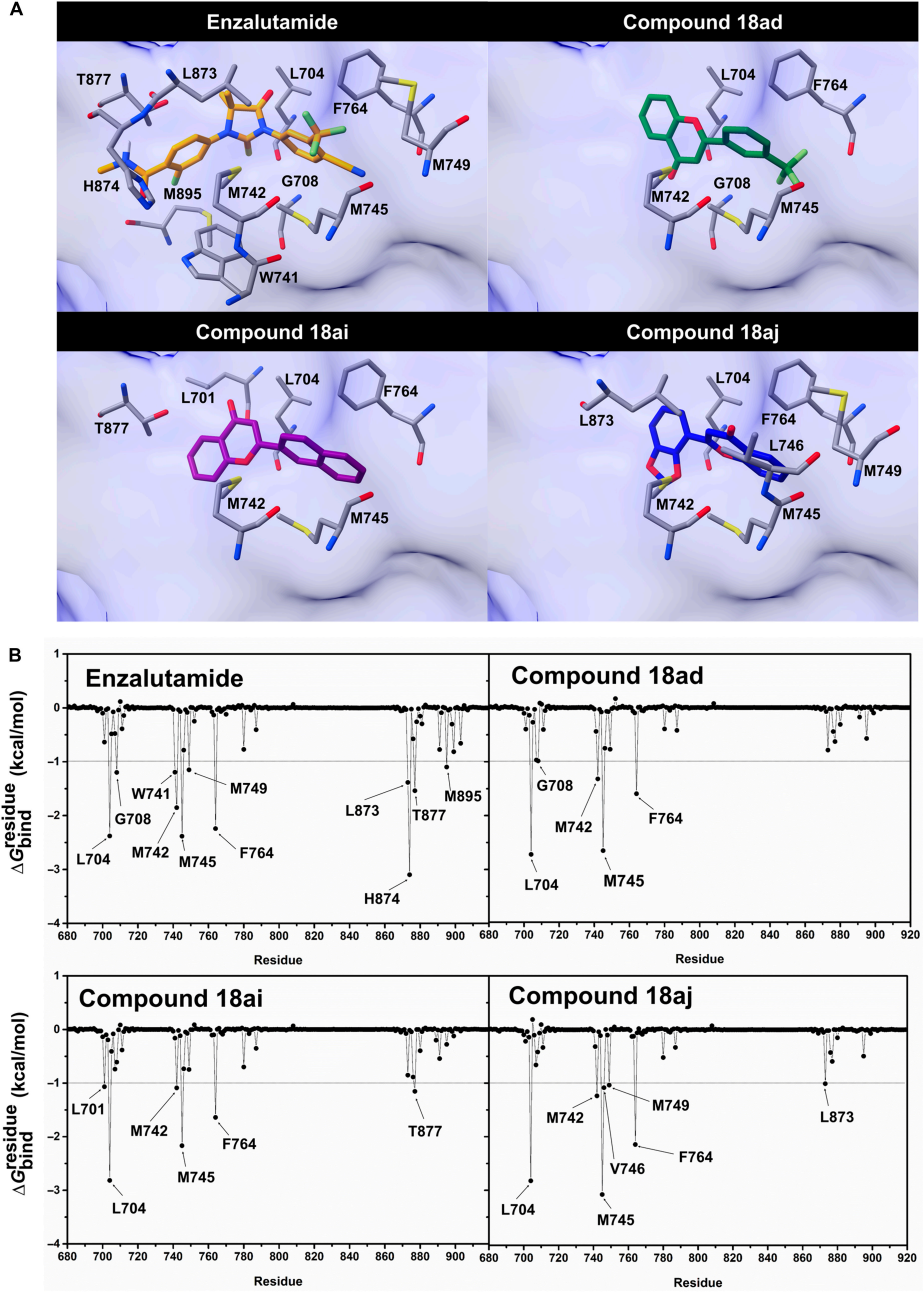
Structural binding patterns and per-residue energy decomposition. (A) Representative three-dimensional (3D) snapshots illustrating the binding conformations of each ligand within the androgen receptor (AR) ligand-binding domain (LBD) binding pocket. (B) Per-residue energy decomposition identifying the specific amino acids mediating the interaction of enzalutamide, 18ad, 18ai, and 18aj toward the AR LBD. Residues with a binding free energy contribution of less than –1.0 kcal/mol are identified as key determinants of complex stability.

To further elucidate the molecular basis of ligand stabilization within the AR LBD, we performed a per-residue energy decomposition analysis ($∆G_{\text{bind}}^{\text{residue}}$) to quantify the energetic contributions of individual amino acid residues. Residues with an energy contribution of less than –1.0 kcal/mol were considered important contributors and are highlighted in Fig. 5. The results revealed that the key interacting residues for compound **18ad** include L704, G708, M742, M745, and F764. Compound **18ai** showed contributions from 6 residues (L701, L704, M742, M745, F764, and T877), while compound **18aj** exhibited 7 key residues (L704, M742, M745, V746, M749, F764, and L873) compared to the binding profile of enzalutamide. It is well established that mutations such as H874Y, F876L, and T877A are drivers of resistance to enzalutamide [59,60]. These structural alterations can promote an agonist-like active conformation of the AR LBD, facilitating coactivator recruitment and downstream transcriptional activation [61]. Because the MD simulations in this study were performed using the wild-type receptor, the dynamic impact of these mutations could not be directly evaluated. To preliminarily explore the structural compatibility of the prioritized compounds with clinically relevant AR variants, we performed additional docking calculations using available mutant AR LBD crystal structures, including H874Y (PDB ID: 2Q7K [62]), T877A (PDB ID: 2AX6 [63]), and W741L (PDB ID: 2AX8 [63]), following the same docking protocol and parameters applied to the wild-type receptor. As shown in Figs. S3 to S5, the predicted binding poses of compounds **18ad**, **18ai**, and **18aj** remained comparable to those observed in the wild-type receptor, with key binding interactions mainly preserved. Regarding the W741L mutation, which is known to alter the binding of bicalutamide [64,65], the $∆G_{\text{bind}}^{\text{residue}}$ analysis indicated that residue W741 did not emerge as a dominant energetic contributor for the 3 compounds. This observation suggests that the W741L mutation may have a less pronounced impact on binding compared with ligands that rely heavily on π-stacking interactions at this position. Taken together, these results highlight promising structural features of the hit compounds; however, further studies are warranted to evaluate their performance against clinically relevant AR mutations.

Beyond the structural modification of natural products, the strategic incorporation of specific functional groups represents a widely applied medicinal chemistry strategy to optimize molecular interactions and physicochemical properties [66]. Among these, the trifluoromethyl (–CF_3_) moiety has attracted considerable attention because of its strong electron-withdrawing character and steric influence, which can modulate intermolecular interactions such as halogen bonding and electrostatic contacts. In addition, fluorinated substituents are frequently associated with enhanced metabolic stability and modified lipophilicity, factors that may influence membrane permeability and pharmacokinetic behavior [67]. Such features have been reported to support ligand recognition within steroid hormone receptors, including the AR, estrogen receptor, glucocorticoid receptor, and progesterone receptor [68,69]. In the present study, compound **18ad**, which bears a trifluoromethyl group analogous to that found in enzalutamide, was prioritized based on computational analysis. The compound displayed favorable drug-likeness characteristics and maintained stable accommodation within the AR ligand-binding pocket during MD simulations. However, although the –CF_3_ substituent may contribute to the observed interaction patterns, the current dataset does not permit definitive structure–activity relationship conclusions, and its specific pharmacological contribution remains to be experimentally validated across a broader flavonoid series. Therefore, compound **18ad** is interpreted here as a structurally promising lead scaffold rather than evidence that –CF_3_ substitution is a key determinant of activity. Collectively, these findings supported the selection of compound **18ad** for subsequent biological evaluation, as described in the following section.

### Effect of compound 18ad on PCa cell viability

AR antagonists, such as bicalutamide and enzalutamide, were developed to specifically attenuate AR activity, forming a cornerstone of therapeutic strategies aimed at preventing PCa proliferation and disease progression [70]. Unfortunately, these drugs can induce adverse effects that promote relapse or even disease advancement, leading to more severe and potentially lethal outcomes due to the development of drug resistance [71]. Thus, inhibiting PCa proliferation remains a significant challenge in clinical practice, as abnormal AR signaling continues to drive the overgrowth of PCa cells.

While the anti-PCa activity of compound **18ad** has not been previously reported, this study investigated its potency using 2 well-established PCa models with distinct AR status: LNCaP (androgen-dependent, AR-positive) and PC-3 (androgen-independent, AR-null) cells. The selection of these complementary cell lines allowed a preliminary assessment of AR-dependent versus AR-independent cellular responses, thereby helping to distinguish pathway-specific effects from general cytotoxicity. Cells were treated with serial dilutions (3.125 to 100 μM) or vehicle control (0.2% DMSO), and viability was evaluated using the MTS assay. Both 24- and 48-h treatment durations were included to evaluate early cellular responses and sustained antiproliferative effects, as modulation of AR signaling and downstream transcriptional programs may require extended exposure to produce measurable changes in cell viability.

**Fig. 6.**
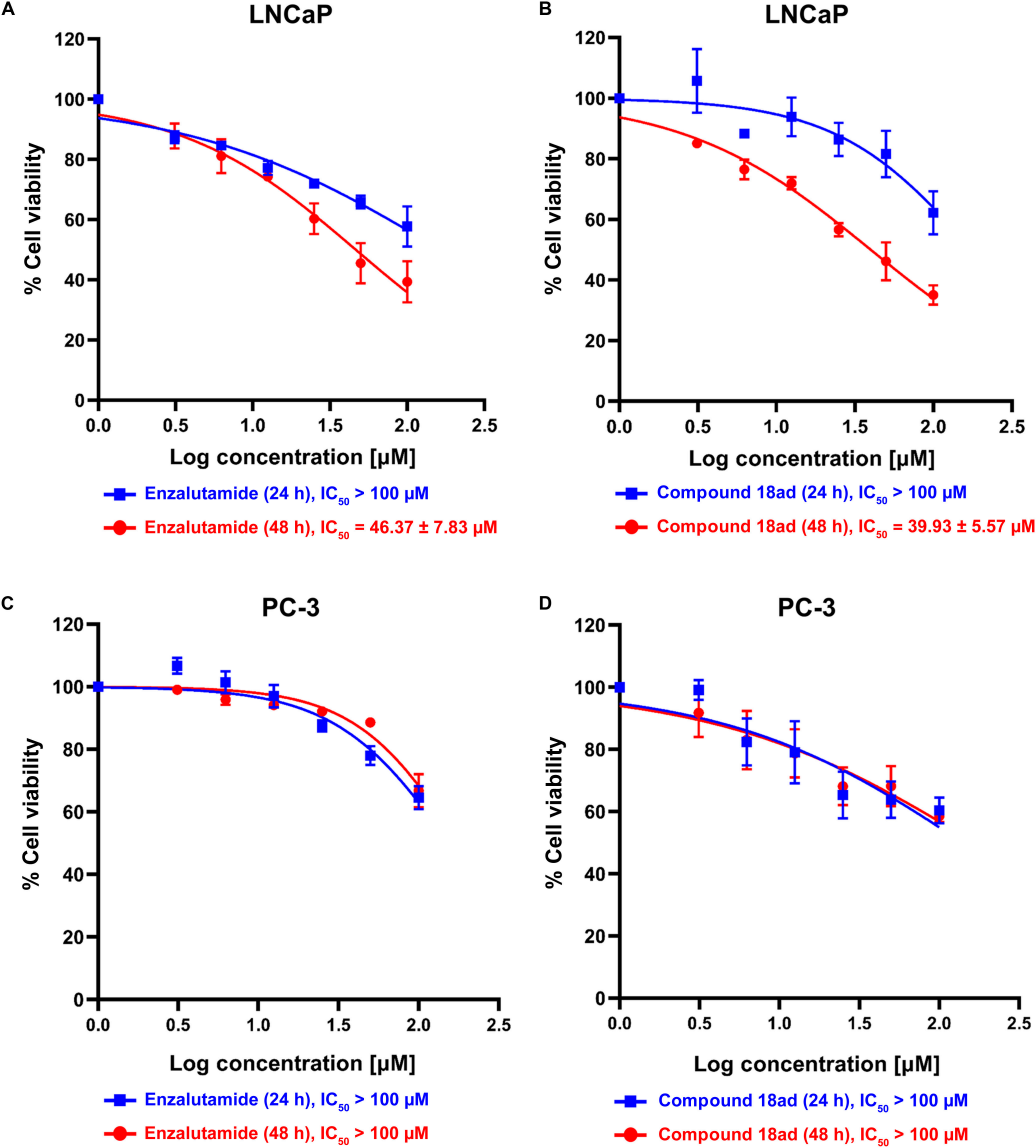
Inhibitory effect of compound 18ad on prostate cancer (PCa) cell viability. LNCaP (A and B) and PC-3 (C and D) cells were treated with enzalutamide or compound 18ad at the indicated concentrations for 24 or 48 h. Panels (A) and (C) show the effects of enzalutamide on LNCaP and PC-3 cells, respectively, whereas panels (B) and (D) show the effects of compound 18ad on LNCaP and PC-3 cells. Cell viability was determined using the MTS assay and normalized to the vehicle-treated control. Data are presented as means ± standard error of the mean (SEM) from 3 independent experiments performed in triplicate.

As shown in Fig. 6, compound **18ad** significantly reduced LNCaP cell growth in a dose- and time-dependent manner at both 24 and 48 h. In contrast, no marked reduction in cell viability was observed in AR-null PC-3 cells (Fig. 6C and D), suggesting that the antiproliferative activity of **18ad** may be associated with AR signaling modulation. It should be noted that the half-maximal inhibitory concentration (IC_50_) values could not be calculated for PC-3 cells because compound treatment did not reduce cell viability below 50% within the tested concentration range (up to 100 μM). At 48 h, the IC_50_ values observed for compound **18ad** (39.93 ± 5.57 μM) fall within the same order of magnitude as those obtained for enzalutamide (46.37 ± 7.83 μM) under the present assay conditions. This similarity reflects the experimental context rather than equivalent pharmacological potency, as cell-based responses to AR antagonists may vary depending on assay design and exposure duration. However, the comparable activity profile relative to the reference drug supports the classification of **18ad** as a promising early-stage hit. These values are consistent with previously reported micromolar activities for flavonoid-based AR modulators, such as alpinumisoflavone, which exhibit inhibition of LNCaP and C4-2 cells within similar concentration ranges [12]. Consistent with this observation, semisynthetic flavonoid scaffolds have demonstrated micromolar-range antiproliferative activity across diverse cancer models, supporting the feasibility of this chemical class as a starting point for further optimization [72,73]. Although compound **18ad** was previously reported to show limited activity against human breast and liver cancer cell lines at high concentrations [25], our findings indicate preferential inhibition of androgen-responsive PCa cells under the conditions tested. Nevertheless, given the moderate micromolar potency observed in this initial screening study, further evaluation in AR-variant-expressing models, nonmalignant epithelial cells, and expanded safety assays is necessary to define selectivity and therapeutic potential.

### Effect of compound 18ad on AR and PSA expression in PCa cells

The expression of the AR is a primary driver of PCa growth and progression. Specifically, the transcription of the *PSA* gene, a critical biomarker for PCa, is directly regulated by AR transcriptional activity. The activation of the AR/PSA axis subsequently supports the progression of PCa-derived malignancies [74]. We performed reverse transcription-qPCR to investigate whether the growth suppression induced by compound **18ad** (at 20, 40, and 60 μM) was associated with the inhibition of AR and its primary downstream target, *PSA*.

**Fig. 7.**
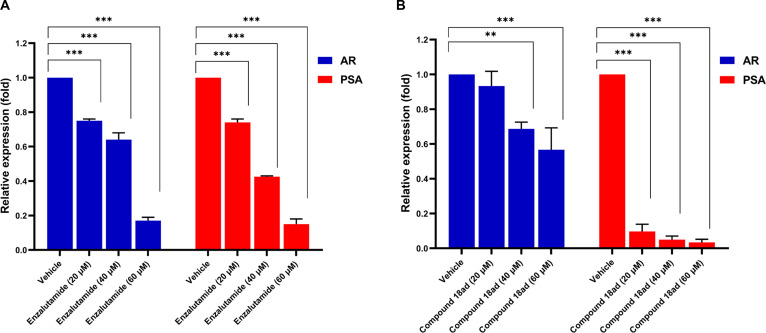
Effect of compound 18ad on *AR* and *PSA* mRNA expression in LNCaP cells. Treatment with (A) enzalutamide and (B) compound 18ad significantly suppressed the transcript levels of *AR* and *PSA* in LNCaP cells compared to the vehicle control. Relative mRNA expression levels (fold change) were normalized to *β-actin*, with the control group set to 1.0. Data are presented as means ± standard error of the mean (SEM) of 3 independent experiments performed in triplicate. **P < 0.01, ***P < 0.001 versus control.

As shown in Fig. 7, compound **18ad** markedly reduced *AR* mRNA expression levels and significantly down-regulated *PSA* expression in LNCaP cells. Consistent with its established mode of action as an AR antagonist, enzalutamide treatment also decreased PSA expression under the same experimental conditions. Because PSA is a direct downstream transcriptional target of AR, changes in *PSA* expression serve as a sensitive indicator of AR functional activity and may reflect alterations in signaling more dynamically than receptor abundance itself [74]. These findings suggest that compound **18ad** may attenuate AR-driven transcriptional programs rather than inducing nonspecific cytotoxic effects. Aligning with previous reports, suppression of AR/PSA signaling has been related to reduced proliferation and invasive potential in PCa cells [12,75]. However, the present study evaluates AR pathway modulation primarily at the transcript level, and mRNA expression does not always directly correlate with protein abundance due to posttranslational regulation and protein stability. Therefore, while the observed reduction in *PSA* supports functional disruption of AR signaling, additional studies, including protein-level analyses and functional reporter assays, are required to define the molecular mechanism of action of compound **18ad**.

## Conclusion

In summary, this study identifies compound **18ad** as a promising nonsteroidal AR antagonist scaffold through an integrated computational–experimental workflow. Structure-based virtual screening combined with physicochemical profiling highlighted compounds **18ad**, **18ai**, and **18aj** as top candidates with favorable predicted docking scores and drug-likeness properties. Subsequent MD simulations and free energy analyses revealed stable accommodation of these compounds within the AR LBD. Biological validation further showed that compound **18ad**, characterized by a trifluoromethyl-substituted flavone, selectively suppresses the proliferation of androgen-dependent LNCaP cells. This antiproliferative effect is associated with modulation of the AR signaling axis, as reflected by decreased *AR* and *PSA* transcript levels. Although the observed micromolar potency reflects an early stage of hit identification, the consistent cellular response together with stable binding behavior supports the potential of this scaffold as a starting point for structure-guided optimization.

Overall, these findings expand the chemical space of nonsteroidal AR-targeting ligands and provide new insight into the activity of fluorinated flavone derivatives in PCa models. Rather than representing a higher-affinity alternative to established AR antagonists, compound **18ad** should be viewed as a structurally promising lead scaffold that could be further optimized to enhance binding efficiency and pharmacological performance. Future investigations should focus on addressing key limitations identified in this study, including evaluation against clinically relevant AR variants (e.g., AR-V7 and resistance-associated mutations), assessment of selectivity in nonmalignant epithelial models, and expanded toxicity profiles. In addition, protein-level validation, functional AR reporter assays, and hit-to-lead optimization are essential to further elucidate the mechanism of action and advance this scaffold within the PCa drug discovery pipeline.

## Data Availability

The datasets used and/or analyzed during the current study are available from the corresponding author on reasonable request.
