# Supplementary material for: Identification of a Novel Trifluoromethyl-Bearing Flavonoid as a Promising Androgen Receptor Antagonist: Structure-Based Virtual Screening and In Vitro Study
Source: Comput Struct Biotechnol J. 2026 Apr 13;35(1):0038. doi: 10.34133/csbj.0038 (PMC13082672; doi:10.34133/csbj.0038)
Supplement: Supplementary file 1 — Tables S1 to S6 Figs. S1 to S5 [file csbj.0038.f1.docx]

**Supplementary Materials**

**Identification of a novel trifluoromethyl-bearing flavonoid as a promising androgen receptor antagonist: Structure-based virtual screening and *in vitro* study**

Phakkhathorn Tadasee^1^, Tanatorn Khotavivattana^2^, Kulathida Chaithirayanon^3^,
Suvichada Assawakosri^1^ and Bodee Nutho^1*^

*^1^Department of Pharmacology, Faculty of Science, Mahidol University, Bangkok 10400, Thailand*

*^2^Center of Excellence in Natural Product, Department of Chemistry, Faculty of Science, Chulalongkorn University, Bangkok, 10330, Thailand*

*^3^Department of Anatomy, Faculty of Science, Mahidol University, Bangkok 10400, Thailand*

^*^Corresponding author. BN Fax: +662-354-7157; Tel: +662-201-5651

E-mail address: bodee.nut@mahidol.ac.th

**Table S1.** Chemical structures of 89 flavonoid derivatives.





| **Compound** | **R^1^** | **R^2^** | **R^3^** | **R^4^** | **R^5^** | **R^6^** | **R^7^** | **R^8^** | **R^9^** |
| --- | --- | --- | --- | --- | --- | --- | --- | --- | --- |
| 1a | H | OH | H | H | H | H | H | H | H |
| 1b | H | H | OH | H | H | H | H | H | H |
| 1c | OH | OH | H | H | H | H | H | H | H |
| 1d | H | OH | H | OH | H | H | H | H | H |
| 1e | H | OH | OH | OH | H | H | H | H | H |
| 1f | H | OH | H | OH | OH | H | OH | OH | H |
| 1g | H | H | Me | H | H | H | H | H | H |
| 2a | H | OMe | H | H | H | H | H | H | H |
| 2b | H | H | OMe | H | H | H | H | H | H |
| 2c | OMe | OMe | H | H | H | H | H | H | H |
| 2d | -OCH_2_O- | -OCH_2_O- | H | H | H | H | H | H | H |
| 2e | H | OMe | H | OH | H | H | H | H | H |
| 2f | H | OBn | H | OH | H | H | H | H | H |
| 2g | H | OMe | H | OMe | H | H | H | H | H |
| 2h | H | OEt | H | OH | H | H | H | H | H |
| 2i | H | OEt | H | OEt | H | H | H | H | H |
| 2j | H | OCH_4_CH=CH_2_ | H | OH | H | H | H | H | H |
| 2k | H | OCH_2_CH(OH)CH_2_OH | H | OH | H | H | H | H | H |
| 2l | H | OMe | OMe | OH | H | H | H | H | H |
| 3a | H | OEt | H | OMe | H | H | H | H | H |
| 3b | H | OCH_4_CH=CH_2_ | H | OMe | H | H | H | H | H |
| 3c | H | OCH_2_CH=C(CH_3_)_2_ | H | OMe | H | H | H | H | H |
| 4 | NO_2_ | OMe | H | OH | H | H | H | H | H |
| 5a | H | OPp | H | H | H | H | H | H | H |
| 5b | H | H | OPp | H | H | H | H | H | H |
| 5c | H | OPp | OPp | OPp | H | H | H | H | H |
| 5d | H | OPp | OPp | OH | H | H | H | H | H |
| 5e | H | OAc | OAc | OAc | H | H | H | H | H |
| 6a | Br | OH | Br | OH | H | H | H | H | H |
| 6b | Br | OH | OH | OH | H | H | H | H | H |
| 7 | Br | OMe | Br | OMe | H | H | H | H | H |
| 8 | Br | OPp | OPp | OPp | H | H | H | H | H |
| 9 | OH | OH | H | H | Cl | H | H | H | H |
| 10a | H | NO_2_ | OH | NO_2_ | H | H | H | H | H |
| 10b | NO_2_ | OH | H | OH | H | H | H | H | H |
| 10c | NO_2_ | OH | NO_2_ | OH | H | H | H | H | H |
| 11 | NH_2_ | OH | H | OH | H | H | H | H | H |
| 12 | NHAc | OH | H | OH | H | H | H | H | H |
| 14a | H | OMe | OMe | OMe | H | H | H | H | H |
| 14b | H | OMe | OMe | OMe | H | H | H | Br | H |
| 14c | H | OMe | OMe | OMe | H | H | H | NO_2_ | H |
| 15 | H | OMe | OMe | OMe | H | H | H | NH_2_ | H |
| 16a | H | OMe | OMe | OH | H | H | H | Br | H |
| 16b | H | OMe | OMe | OH | H | H | H | NO_2_ | H |
| 18aa | H | H | H | H | H | F | H | H | H |
| 18ab | H | H | H | H | H | H | OMe | H | H |
| 18ac | H | H | H | H | H | H | NO­_2_ | H | H |
| 18ad | H | H | H | H | H | H | CF_3_ | H | H |
| 18ae | H | H | H | H | H | H | H | OMe | H |
| 18af | H | H | H | H | H | H | H | Cl | H |
| 18ag | H | H | H | H | H | H | H | Br | H |
| 18ah | H | H | H | H | H | H | H | C_6_H_5_ | H |
| 18ai | H | H | H | H | H | H | -CH=CH-H=CH- | -CH=CH-H=CH- | H |
| 18aj | H | H | H | H | H | -OCH_2_O- | -OCH_2_O- | H | H |
| 18ak | H | OMe | H | OMe | H | H | H | OMe | H |
| 18al | H | OMe | H | OMe | H | H | OMe | H | H |
| 18am | H | OMe | H | OMe | H | OMe | H | H | H |
| 18an | H | OMe | H | OMe | H | H | OMe | OMe | H |
| 18ao | H | OMe | H | OMe | H | Br | H | OMe | OMe |
| 18ap | H | OMe | H | OMe | H | H | F | OMe | H |
| 18aq | H | OMe | H | OMe | H | H | OMe | F | H |
| 18ar | H | OMe | H | OMe | H | OMe | H | OMe | H |
| 18as | H | OMe | H | OMe | H | OMe | H | H | OMe |
| 18at | H | OMe | H | OMe | H | OMe | OMe | OMe | H |
| 18au | H | OMe | H | OMe | H | H | OMe | OMe | OMe |
| 18av | H | OMe | H | OMe | H | H | NO­_2_ | H | H |
| 18aw | H | OMe | H | OMe | H | H | Br | H | H |
| 18ax | H | OMe | H | OMe | H | H | H | OCF_3_ | H |
| 18ay | H | OMe | H | OMe | H | H | H | Cl | H |
| 18az | H | OMe | H | OMe | H | H | H | SMe | H |
| 18ba | H | OMe | H | OMe | H | H | F | F | H |
| 18bb | H | OMe | OMe | H | H | H | H | H | H |
| 18bc | H | H | F | H | H | H | H | H | H |
| 18bd | H | F | H | H | H | H | H | H | H |
| 18be | H | Cl | H | H | H | H | H | H | H |
| 18bf | H | H | NO_2_ | H | H | H | H | H | H |
| 18bg | H | Br | H | H | H | H | H | H | H |
| 18bh | H | H | Br | H | H | H | H | H | H |
| 18bi | H | Me | Cl | H | H | H | H | H | H |
| 18bj | NO_2_ | H | Me | H | H | H | H | H | H |
| 18bk | Cl | H | Cl | H | H | H | H | H | H |
| 18bl | Br | H | Br | H | H | H | H | H | H |
| 18bm | H | OMe | H | H | H | H | H | Br | H |
| 18bn | H | H | Me | H | H | H | H | Br | H |
| 18bo | Br | H | Br | H | H | H | OMe | OMe | H |
| 19a | H | OH | H | OH | H | Br | H | OH | OH |
| 19b | H | OH | H | OH | H | H | H | OCF_3_ | H |
| 19c | H | OMe | H | OH | H | H | H | SMe | H |
| 19d | H | OH | H | OH | H | H | H | SMe | H |

**Table S2.** Chemical structures of 23 chalcone derivatives.





| **Compound** | **R^1^** | **R^2^** | **R^3^** | **R^4^** | **R^5^** | **R^6^** | **R^7^** | **R^8^** | **R^9^** |
| --- | --- | --- | --- | --- | --- | --- | --- | --- | --- |
| C1 | OCH_3_ | H | OCH_3_ | H | H | H | NO_2_ | H | H |
| C2 | OCH_3_ | OCH_3_ | OCH_3_ | H | H | H | NO_2_ | H | H |
| C3 | H | OCH_3_ | OCH_3_ | H | H | H | H | NO_2_ | H |
| C4 | OCH_3_ | H | H | H | H | NO_2_ | H | H | H |
| C5 | OCH_3_ | H | H | H | OCH_3_ | NO_2_ | H | NO_2_ | H |
| C6 | H | OCH_3_ | OCH_3_ | OCH_3_ | H | H | NO_2_ | H | H |
| C7 | OCH_3_ | H | OCH_3_ | H | OCH_3_ | NO_2_ | H | H | H |
| C8 | OCH_3_ | H | OCH_3_ | H | OCH_3_ | H | H | NO_2_ | H |
| C9 | OCH_3_ | H | OCH_3_ | H | OCH_3_ | CF_3_ | H | H | H |
| C10 | OCH_3_ | H | OCH_3_ | H | OCH_3_ | H | CF_3_ | H | H |
| C11 | OCH_3_ | H | OCH_3_ | H | OCH_3_ | H | H | CF_3_ | H |
| C12 | OCH_3_ | H | OCH_3_ | H | OCH_3_ | H | H | CN | H |
| C13 | OCH_3_ | H | OCH_3_ | H | OCH_3_ | H | CN | H | H |
| C14 | H | OCH_3_ | OCH_3_ | H | H | H | H | CN | H |
| C15 | OCH_3_ | H | H | H | H | H | H | CN | H |
| C16 | H | OCH_3_ | H | H | H | H | H | CN | H |
| C17 | OCH_3_ | H | H | H | H | H | CN | H | H |
| C18 | OCH_3_ | H | OCH_3_ | H | H | H | CN | H | H |
| C19 | OCH_3_ | H | H | OCH_3_ | H | H | CN | H | H |
| C20 | OCH_3_ | H | H | H | OCH_3_ | H | CN | H | H |
| C21 | H | OCH_3_ | H | OCH_3_ | H | H | CN | H | H |
| SU086 | OCH_3_ | H | OCH_3_ | H | OCH_3_ | H | NO_2_ | H | H |
| BSU086 | Biotin-SS-COOH | H | OCH_3_ | H | OCH_3_ | H | NO_2_ | H | H |

**Table S3.** Molecular structures of in-house compounds in SMILES format.

| **Compound** | **SMILES** |
| --- | --- |
| 1a | O=C1C2=CC=C(O)C=C2OC(C3=CC=CC=C3)=C1 |
| 1b | O=C1C2=CC(O)=CC=C2OC(C3=CC=CC=C3)=C1 |
| 1c | O=C1C2=CC=C(O)C(O)=C2OC(C3=CC=CC=C3)=C1 |
| 1d | O=C1C2=C(O)C=C(O)C=C2OC(C3=CC=CC=C3)=C1 |
| 1e | O=C1C2=C(O)C(O)=C(O)C=C2OC(C3=CC=CC=C3)=C1 |
| 1f | O=C1C2=C(O)C=C(O)C=C2OC(C3=CC=C(O)C(O)=C3)=C1O |
| 1g | O=C1C2=CC(C)=CC=C2OC(C3=CC=CC=C3)=C1 |
| 2a | O=C1C2=CC=C(OC)C=C2OC(C3=CC=CC=C3)=C1 |
| 2b | O=C1C2=CC(OC)=CC=C2OC(C3=CC=CC=C3)=C1 |
| 2c | O=C1C2=CC=C(OC)C(OC)=C2OC(C3=CC=CC=C3)=C1 |
| 2d | O=C1C2=CC=C3C(OCO3)=C2OC(C4=CC=CC=C4)=C1 |
| 2e | O=C1C2=C(O)C=C(OC)C=C2OC(C3=CC=CC=C3)=C1 |
| 2f | O=C1C2=C(O)C=C(OCC3=CC=CC=C3)C=C2OC(C4=CC=CC=C4)=C1 |
| 2g | O=C1C2=C(OC)C=C(OC)C=C2OC(C3=CC=CC=C3)=C1 |
| 2h | O=C1C2=C(O)C=C(OCC)C=C2OC(C3=CC=CC=C3)=C1 |
| 2i | O=C1C2=C(OCC)C=C(OCC)C=C2OC(C3=CC=CC=C3)=C1 |
| 2j | O=C1C2=C(O)C=C(OCC=C)C=C2OC(C3=CC=CC=C3)=C1 |
| 2k | O=C1C2=C(O)C=C(OCC(O)CO)C=C2OC(C3=CC=CC=C3)=C1 |
| 2l | O=C1C2=C(O)C(OC)=C(OC)C=C2OC(C3=CC=CC=C3)=C1 |
| 3a | O=C1C2=C(OC)C=C(OCC)C=C2OC(C3=CC=CC=C3)=C1 |
| 3b | O=C1C2=C(OC)C=C(OCC=C)C=C2OC(C3=CC=CC=C3)=C1 |
| 3c | O=C1C2=C(OC)C=C(OC/C=C(C)/C)C=C2OC(C3=CC=CC=C3)=C1 |
| 4 | O=C1C2=C(O)C=C(OC)C([N+]([O-])=O)=C2OC(C3=CC=CC=C3)=C1 |
| 5a | O=C1C2=CC=C(OC(CC)=O)C=C2OC(C3=CC=CC=C3)=C1 |
| 5b | O=C1C2=CC(OC(CC)=O)=CC=C2OC(C3=CC=CC=C3)=C1 |
| 5c | O=C1C2=C(OC(CC)=O)C(OC(CC)=O)=C(OC(CC)=O)C=C2OC(C3=CC=CC=C3)=C1 |
| 5d | O=C1C2=C(O)C(OC(CC)=O)=C(OC(CC)=O)C=C2OC(C3=CC=CC=C3)=C1 |
| 5e | O=C1C2=C(OC(C)=O)C(OC(C)=O)=C(OC(C)=O)C=C2OC(C3=CC=CC=C3)=C1 |
| 6a | O=C1C2=C(O)C(Br)=C(O)C(Br)=C2OC(C3=CC=CC=C3)=C1 |
| 6b | O=C1C2=C(O)C(O)=C(O)C(Br)=C2OC(C3=CC=CC=C3)=C1 |
| 7 | O=C1C2=C(OC)C(Br)=C(OC)C(Br)=C2OC(C3=CC=CC=C3)=C1 |
| 8 | O=C1C2=C(OC(CC)=O)C(OC(CC)=O)=C(OC(CC)=O)C(Br)=C2OC(C3=CC=CC=C3)=C1 |
| 9 | O=C1C2=CC=C(O)C(O)=C2OC(C3=CC=CC=C3)=C1Cl |
| 10a | O=C1C2=C([N+]([O-])=O)C(O)=C([N+]([O-])=O)C=C2OC(C3=CC=CC=C3)=C1 |
| 10b | O=C1C2=C(O)C=C(O)C([N+]([O-])=O)=C2OC(C3=CC=CC=C3)=C1 |
| 10c | O=C1C2=C(O)C([N+]([O-])=O)=C(O)C([N+]([O-])=O)=C2OC(C3=CC=CC=C3)=C1 |
| 11 | O=C1C2=C(O)C=C(O)C(N)=C2OC(C3=CC=CC=C3)=C1 |
| 12 | O=C1C2=C(O)C=C(O)C(NC(C)=O)=C2OC(C3=CC=CC=C3)=C1 |
| 14a | O=C1C2=C(OC)C(OC)=C(OC)C=C2OC(C3=CC=CC=C3)=C1 |
| 14b | O=C1C2=C(OC)C(OC)=C(OC)C=C2OC(C3=CC=C(Br)C=C3)=C1 |
| 14c | O=C1C2=C(OC)C(OC)=C(OC)C=C2OC(C3=CC=C([N+]([O-])=O)C=C3)=C1 |
| 15 | O=C1C2=C(OC)C(OC)=C(OC)C=C2OC(C3=CC=C(N)C=C3)=C1 |
| 16a | O=C1C2=C(O)C(OC)=C(OC)C=C2OC(C3=CC=C(Br)C=C3)=C1 |
| 16b | O=C1C2=C(O)C(OC)=C(OC)C=C2OC(C3=CC=C([N+]([O-])=O)C=C3)=C1 |
| 18aa | O=C1C2=CC=CC=C2OC(C3=CC=CC=C3F)=C1 |
| 18ab | O=C1C2=CC=CC=C2OC(C3=CC=CC(OC)=C3)=C1 |
| 18ac | O=C1C2=CC=CC=C2OC(C3=CC=CC([N+]([O-])=O)=C3)=C1 |
| 18ad | O=C1C2=CC=CC=C2OC(C3=CC=CC(C(F)(F)F)=C3)=C1 |
| 18ae | O=C1C2=CC=CC=C2OC(C3=CC=C(OC)C=C3)=C1 |
| 18af | O=C1C2=CC=CC=C2OC(C3=CC=C(Cl)C=C3)=C1 |
| 18ag | O=C1C2=CC=CC=C2OC(C3=CC=C(Br)C=C3)=C1 |
| 18ah | O=C1C2=CC=CC=C2OC(C3=CC=C(C4=CC=CC=C4)C=C3)=C1 |
| 18ai | O=C1C2=CC=CC=C2OC(C3=CC=C(C=CC=C4)C4=C3)=C1 |
| 18aj | O=C1C2=CC=CC=C2OC(C3=CC=CC4=C3OCO4)=C1 |
| 18ak | O=C1C2=C(OC)C=C(OC)C=C2OC(C3=CC=C(OC)C=C3)=C1 |
| 18al | O=C1C2=C(OC)C=C(OC)C=C2OC(C3=CC=CC(OC)=C3)=C1 |
| 18am | O=C1C2=C(OC)C=C(OC)C=C2OC(C3=CC=CC=C3OC)=C1 |
| 18an | O=C1C2=C(OC)C=C(OC)C=C2OC(C3=CC=C(OC)C(OC)=C3)=C1 |
| 18ao | O=C1C2=C(OC)C=C(OC)C=C2OC(C3=CC(OC)=C(OC)C=C3Br)=C1 |
| 18ap | O=C1C2=C(OC)C=C(OC)C=C2OC(C3=CC=C(OC)C(F)=C3)=C1 |
| 18aq | O=C1C2=C(OC)C=C(OC)C=C2OC(C3=CC=C(F)C(OC)=C3)=C1 |
| 18ar | O=C1C2=C(OC)C=C(OC)C=C2OC(C3=CC=C(OC)C=C3OC)=C1 |
| 18as | O=C1C2=C(OC)C=C(OC)C=C2OC(C3=CC(OC)=CC=C3OC)=C1 |
| 18at | O=C1C2=C(OC)C=C(OC)C=C2OC(C3=CC=C(OC)C(OC)=C3OC)=C1 |
| 18au | O=C1C2=C(OC)C=C(OC)C=C2OC(C3=CC(OC)=C(OC)C(OC)=C3)=C1 |
| 18av | O=C1C2=C(OC)C=C(OC)C=C2OC(C3=CC=CC([N+]([O-])=O)=C3)=C1 |
| 18aw | O=C1C2=C(OC)C=C(OC)C=C2OC(C3=CC=CC(Br)=C3)=C1 |
| 18ax | O=C1C2=C(OC)C=C(OC)C=C2OC(C3=CC=C(OC(F)(F)F)C=C3)=C1 |
| 18ay | O=C1C2=C(OC)C=C(OC)C=C2OC(C3=CC=C(Cl)C=C3)=C1 |
| 18az | O=C1C2=C(OC)C=C(OC)C=C2OC(C3=CC=C(SC)C=C3)=C1 |
| 18ba | O=C1C2=C(OC)C=C(OC)C=C2OC(C3=CC=C(F)C(F)=C3)=C1 |
| 18bb | O=C1C2=CC(OC)=C(OC)C=C2OC(C3=CC=CC=C3)=C1 |
| 18bc | O=C1C2=CC(F)=CC=C2OC(C3=CC=CC=C3)=C1 |
| 18bd | O=C1C2=CC=C(F)C=C2OC(C3=CC=CC=C3)=C1 |
| 18be | O=C1C2=CC=C(Cl)C=C2OC(C3=CC=CC=C3)=C1 |
| 18bf | O=C1C2=CC([N+]([O-])=O)=CC=C2OC(C3=CC=CC=C3)=C1 |
| 18bg | O=C1C2=CC=C(Br)C=C2OC(C3=CC=CC=C3)=C1 |
| 18bh | O=C1C2=CC(Br)=CC=C2OC(C3=CC=CC=C3)=C1 |
| 18bi | O=C1C2=CC(Cl)=C(C)C=C2OC(C3=CC=CC=C3)=C1 |
| 18bj | O=C1C2=CC(C)=CC([N+]([O-])=O)=C2OC(C3=CC=CC=C3)=C1 |
| 18bk | O=C1C2=CC(Cl)=CC(Cl)=C2OC(C3=CC=CC=C3)=C1 |
| 18bl | O=C1C2=CC(Br)=CC(Br)=C2OC(C3=CC=CC=C3)=C1 |
| 18bm | O=C1C2=CC=C(OC)C=C2OC(C3=CC=C(Br)C=C3)=C1 |
| 18bn | O=C1C2=CC(C)=CC=C2OC(C3=CC=C(Br)C=C3)=C1 |
| 18bo | O=C1C2=CC(Br)=CC(Br)=C2OC(C3=CC=C(OC)C(OC)=C3)=C1 |
| 19a | O=C1C2=C(O)C=C(O)C=C2OC(C3=CC(O)=C(O)C=C3Br)=C1 |
| 19b | O=C1C2=C(O)C=C(O)C=C2OC(C3=CC=C(OC(F)(F)F)C=C3)=C1 |
| 19c | O=C1C2=C(O)C=C(OC)C=C2OC(C3=CC=C(SC)C=C3)=C1 |
| 19d | O=C1C2=C(O)C=C(O)C=C2OC(C3=CC=C(SC)C=C3)=C1 |
| BSU086 | O=N(C1=CC(/C=C\C(C2=C(OC)C=C(OC)C=C2OC(CCSSCCNC(CCCCC3C(NC(N4)=O)C4CS3)=O)=O)=O)=CC=C1)=O |
| C1 | O=C(/C=C/C1=CC=CC(N(=O)=O)=C1)C2=CC=C(OC)C=C2OC |
| C2 | O=C(/C=C/C1=CC=CC(N(=O)=O)=C1)C2=CC=C(OC)C(OC)=C2OC |
| C3 | O=C(/C=C/C1=CC=C(N(=O)=O)C=C1)C2=CC=C(OC)C(OC)=C2 |
| C4 | O=C(/C=C/C1=CC=CC=C1N(=O)=O)C2=CC=CC=C2OC |
| C5 | O=C(/C=C/C1=CC=C(N(=O)=O)C=C1N(=O)=O)C2=C(OC)C=CC=C2OC |
| C6 | O=C(/C=C/C1=CC=CC(N(=O)=O)=C1)C2=CC(OC)=C(OC)C(OC)=C2 |
| C7 | O=C(/C=C/C1=CC=CC=C1N(=O)=O)C2=C(OC)C=C(OC)C=C2OC |
| C8 | O=C(/C=C/C1=CC=C(N(=O)=O)C([H])=C1)C2=C(OC)C=C(OC)C=C2OC |
| C9 | O=C(/C=C/C1=CC=CC=C1C(F)(F)F)C2=C(OC)C=C(OC)C=C2OC |
| C10 | O=C(/C=C/C1=CC=CC(C(F)(F)F)=C1)C2=C(OC)C=C(OC)C=C2OC |
| C11 | O=C(/C=C/C1=CC=C(C(F)(F)F)C=C1)C2=C(OC)C=C(OC)C=C2OC |
| C12 | O=C(/C=C/C1=CC=C(C#N)C=C1)C2=C(OC)C=C(OC)C=C2OC |
| C13 | O=C(/C=C/C1=CC=CC(C#N)=C1)C2=C(OC)C=C(OC)C=C2OC |
| C14 | O=C(C1=CC(OC)=C(C=C1)OC)/C=C/C2=CC=C(C=C2)C#N |
| C15 | O=C(/C=C/C1=CC=C(C#N)C=C1)C2=CC=CC=C2OC |
| C16 | O=C(/C=C/C1=CC=C(C#N)C=C1)C2=CC=CC(OC)=C2 |
| C17 | O=C(/C=C/C1=CC=CC(C#N)=C1)C2=CC=CC=C2OC |
| C18 | O=C(/C=C/C1=CC=CC(C#N)=C1)C2=CC=C(OC)C=C2OC |
| C19 | O=C(/C=C/C1=CC=CC(C#N)=C1)C2=CC(OC)=CC=C2OC |
| C20 | O=C(/C=C/C1=CC=CC(C#N)=C1)C2=C(OC)C=CC=C2OC |
| C21 | O=C(/C=C/C1=CC=CC(C#N)=C1)C2=CC(OC)=CC(OC)=C2 |
| SU086 | O=C(/C=C/C1=CC(N(=O)=O)=CC=C1)C2=C(OC)C=C(OC)C=C2OC |
| Dihydrotestosterone (DHT) | CC12CCC3C(CCC4CC(=O)CCC34C)C1CCC2O |
| Enzalutamide (Enz) | CNC(=O)C1=C(F)C=C(C=C1)N2C(=S)N(C(=O)C2(C)C)C3=CC(=C(C=C3)C#N)C(F)(F)F |


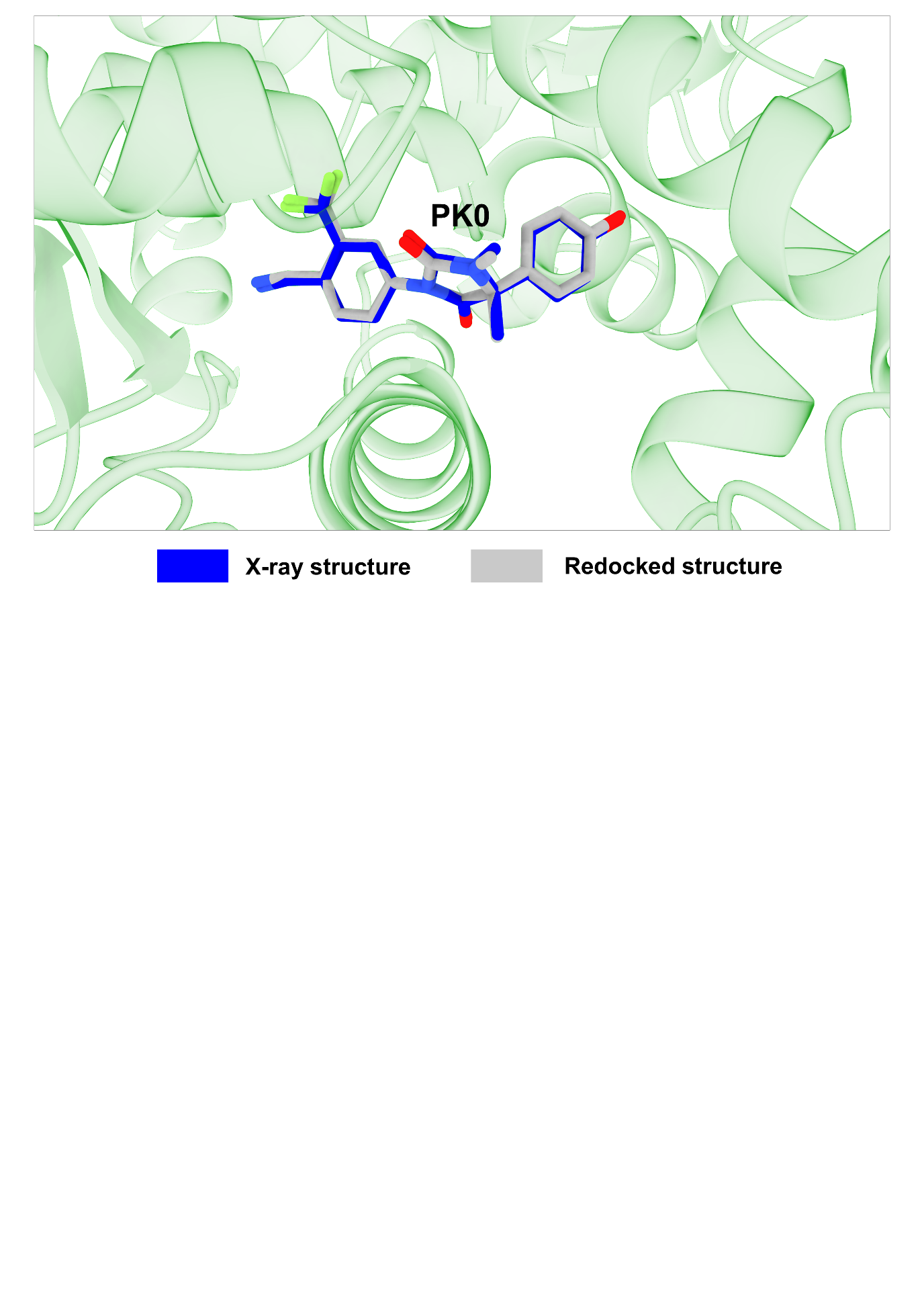


**Figure S1.** Superimposition of the redocked structure of PK0 (grey) onto its X-ray crystallographic pose (blue). The redocking yielded an RMSD of 1.12 Å; values below 2 Å are generally considered satisfactory for validating molecular docking simulations.

**Table S4.** Docking scores of known agonist and antagonist toward the AR LBD (PDB ID: 3V49) obtained via AutoDock Vina scoring function.

| **Compound** | **Docking score (kcal/mol)** |
| --- | --- |
| Agonist | |
| Testosterone | −7.37 |
| Oxandrolone | −6.76 |
| Dihydrotestosterone | −6.40 |
| Stanozolol | −6.08 |
| Antagonist | |
| Nilutamide | −9.09 |
| Bicalutamide | −8.38 |
| Flutamide | −7.88 |
| Enzalutamide | −7.87 |
| Darolutamide | −7.33 |

**Table S5.** Types of interactions of enzalutamide and compounds **18ad**, **18ai**, and **18aj** with the AR LBD.

| Compound | Van der Waals | Conventional Hydrogen Bond | Halogen (Fluorine) | Carbon Hydrogen Bond/π–Donor Hydrogen Bond | π–$\boldsymbol{\sigma}$ | π–π T-shaped/ Amide–π stacked | Alkyl/π–alkyl | π–sulfur |
| --- | --- | --- | --- | --- | --- | --- | --- | --- |
| Enzalutamide | Asn705, Leu707, Gly708, Gln711, Met749, Phe876, Ile899, Val903 | Leu704, Arg752,  His874 | Met745 | Thr877 | Met742 | Trp741, Phe764 | Leu701, Val746, Met787, Leu873 | Met895 |
| 18ad | Leu701, Asn705, Gln711, Trp741, Val746, Leu873, Phe876 | Arg752 | Met745, Phe764 | Gly708, Thr877 | – | – | Leu704, Leu707, Met742, Met749 | Met780, Met787 |
| 18ai | Leu701, Asn705, Gly708, Gln711, Trp741, Met742, Val746, Arg752, Met780, Leu873, Phe876, Phe891, Ile899 | – | – | Thr877 | Met745 | Leu704, Phe764 | Leu707, Met749, Met895 | – |
| 18aj | Leu701, Asn705, Gly708, Gln711, Trp741, Met742, Val746, Arg752, Leu873, Thr877, Met895 | – | – | – | Met745 | Phe764 | Leu704, Leu707, Met749 | Met780, Met787 |

**Table S6.** Evaluation of the physicochemical properties, drug-likeness, lipophilicity, and solubility of 63 compounds using SwissADME software.

| Compound Name | Molecular Weight | H-bond acceptors | H-bond donors | TPSA (Å^2^) | Lipinski’s violations | MLOGP | WLOGP | ESOL Class | Ali Class | Silicos-IT class | Consensus  Log P_o/w_ | Synthetic accessibility | PAINS |
| --- | --- | --- | --- | --- | --- | --- | --- | --- | --- | --- | --- | --- | --- |
| Enzalutamide | 464.44 | 7 | 1 | 108.53 | 0 | 2.73 | 4.80 | Moderately soluble | Moderately soluble | Poorly soluble | 3.80 | 3.17 | 0 |
| 18ad | 290.24 | 5 | 0 | 30.21 | 0 | 3.15 | 5.63 | Moderately soluble | Moderately soluble | Poorly soluble | 4.21 | 3.04 | 0 |
| 18aj | 266.25 | 4 | 0 | 48.67 | 0 | 1.74 | 3.19 | Moderately soluble | Moderately soluble | Moderately soluble | 3 | 3.11 | 0 |
| 18ai | 272.3 | 2 | 0 | 30.21 | 0 | 3.06 | 4.61 | Moderately soluble | Moderately soluble | Poorly soluble | 4.09 | 3.08 | 0 |
| 18bd | 240.23 | 3 | 0 | 30.21 | 0 | 2.67 | 4.02 | Moderately soluble | Moderately soluble | Poorly soluble | 3.56 | 2.79 | 0 |
| 18ac | 267.24 | 4 | 0 | 76.03 | 0 | 2.01 | 3.37 | Moderately soluble | Moderately soluble | Moderately soluble | 2.56 | 2.99 | 0 |
| 1f | 302.24 | 7 | 5 | 131.36 | 0 | -0.56 | 1.99 | Soluble | Soluble | Soluble | 1.23 | 3.23 | 1 |
| 18aa | 240.23 | 3 | 0 | 30.21 | 0 | 2.67 | 4.02 | Moderately soluble | Soluble | Poorly soluble | 3.49 | 2.92 | 0 |
| 18ab | 252.26 | 3 | 0 | 39.44 | 0 | 1.91 | 3.47 | Moderately soluble | Moderately soluble | Poorly soluble | 3.15 | 2.8 | 0 |
| 1d | 254.24 | 4 | 2 | 70.67 | 0 | 1.08 | 2.87 | Moderately soluble | Moderately soluble | Moderately soluble | 2.55 | 2.93 | 0 |
| 18bc | 240.23 | 3 | 0 | 30.21 | 0 | 2.67 | 4.02 | Moderately soluble | Moderately soluble | Poorly soluble | 3.56 | 2.78 | 0 |
| 18af | 256.68 | 2 | 0 | 30.21 | 0 | 2.79 | 4.11 | Moderately soluble | Moderately soluble | Poorly soluble | 3.71 | 2.84 | 0 |
| 18be | 256.68 | 2 | 0 | 30.21 | 0 | 2.79 | 4.11 | Moderately soluble | Moderately soluble | Poorly soluble | 3.78 | 2.87 | 0 |
| 6a | 412.03 | 4 | 2 | 70.67 | 0 | 2.35 | 4.4 | Moderately soluble | Moderately soluble | Poorly soluble | 3.66 | 2.94 | 0 |
| 2e | 268.26 | 4 | 1 | 59.67 | 0 | 1.33 | 3.17 | Moderately soluble | Moderately soluble | Moderately soluble | 2.95 | 3.01 | 0 |
| 2a | 252.26 | 3 | 0 | 39.44 | 0 | 1.91 | 3.47 | Moderately soluble | Moderately soluble | Poorly soluble | 3.24 | 2.93 | 0 |
| 3a | 296.32 | 4 | 0 | 48.67 | 0 | 1.81 | 3.87 | Moderately soluble | Moderately soluble | Poorly soluble | 3.49 | 3.29 | 0 |
| 1g | 236.27 | 2 | 0 | 30.21 | 0 | 2.52 | 3.77 | Moderately soluble | Moderately soluble | Poorly soluble | 3.51 | 2.99 | 0 |
| 1a | 238.24 | 3 | 1 | 50.44 | 0 | 1.66 | 3.17 | Moderately soluble | Moderately soluble | Moderately soluble | 2.84 | 2.87 | 0 |
| 11 | 269.25 | 4 | 3 | 96.69 | 0 | 0.52 | 2.46 | Soluble | Moderately soluble | Moderately soluble | 2.02 | 3 | 0 |
| 3c | 336.38 | 4 | 0 | 48.67 | 0 | 2.42 | 4.81 | Moderately soluble | Moderately soluble | Poorly soluble | 4.27 | 3.54 | 0 |
| 5a | 294.3 | 4 | 0 | 56.51 | 0 | 2.28 | 3.78 | Moderately soluble | Moderately soluble | Poorly soluble | 3.52 | 3.09 | 0 |
| 1e | 270.24 | 5 | 3 | 90.9 | 0 | 0.52 | 2.58 | Moderately soluble | Moderately soluble | Moderately soluble | 2.24 | 3.02 | 1 |
| 19a | 365.13 | 6 | 4 | 111.13 | 0 | 0.61 | 3.04 | Moderately soluble | Moderately soluble | Moderately soluble | 2.33 | 3.18 | 1 |
| 1b | 238.24 | 3 | 1 | 50.44 | 0 | 1.66 | 3.17 | Moderately soluble | Moderately soluble | Moderately soluble | 2.85 | 2.81 | 0 |
| 2b | 252.26 | 3 | 0 | 39.44 | 0 | 1.91 | 3.47 | Moderately soluble | Moderately soluble | Poorly soluble | 3.23 | 2.89 | 0 |
| 18ag | 301.13 | 2 | 0 | 30.21 | 0 | 2.91 | 4.22 | Moderately soluble | Moderately soluble | Poorly soluble | 3.8 | 2.88 | 0 |
| 18bg | 301.13 | 2 | 0 | 30.21 | 0 | 2.91 | 4.22 | Moderately soluble | Moderately soluble | Poorly soluble | 3.88 | 2.96 | 0 |
| 2j | 294.3 | 4 | 1 | 59.67 | 0 | 1.74 | 3.73 | Moderately soluble | Moderately soluble | Poorly soluble | 3.37 | 3.19 | 0 |
| C13 | 263.29 | 3 | 0 | 50.09 | 0 | 2.32 | 3.35 | Soluble | Moderately soluble | Moderately soluble | 3.13 | 2.67 | 0 |
| C14 | 263.29 | 3 | 0 | 50.09 | 0 | 2.32 | 3.35 | Soluble | Moderately soluble | Moderately soluble | 3.13 | 2.67 | 0 |
| C16 | 293.32 | 4 | 0 | 59.32 | 0 | 1.97 | 3.36 | Soluble | Moderately soluble | Moderately soluble | 3.16 | 2.64 | 0 |
| 2d | 266.25 | 4 | 0 | 48.67 | 0 | 1.74 | 3.19 | Moderately soluble | Moderately soluble | Moderately soluble | 3 | 3.15 | 0 |
| 3b | 308.33 | 4 | 0 | 48.67 | 0 | 1.97 | 4.03 | Moderately soluble | Moderately soluble | Poorly soluble | 3.68 | 3.33 | 0 |
| 9 | 288.68 | 4 | 2 | 70.67 | 0 | 1.6 | 3.52 | Moderately soluble | Moderately soluble | Moderately soluble | 2.87 | 2.99 | 1 |
| 2c | 282.29 | 4 | 0 | 48.67 | 0 | 1.57 | 3.48 | Soluble | Soluble | Poorly soluble | 3.04 | 3.22 | 0 |
| C5 | 328.28 | 6 | 0 | 117.94 | 0 | 0.88 | 4.35 | Soluble | Moderately soluble | Moderately soluble | 2.13 | 2.94 | 0 |
| 19b | 338.23 | 8 | 2 | 79.9 | 0 | 1.13 | 5.03 | Moderately soluble | Moderately soluble | Moderately soluble | 3.39 | 3 | 0 |
| 2h | 282.29 | 4 | 1 | 59.67 | 0 | 1.57 | 3.56 | Moderately soluble | Moderately soluble | Poorly soluble | 3.27 | 3.15 | 0 |
| C18 | 323.34 | 5 | 0 | 68.55 | 0 | 1.64 | 3.37 | Soluble | Moderately soluble | Moderately soluble | 3.11 | 3.08 | 0 |
| 18am | 312.32 | 5 | 0 | 57.9 | 0 | 1.25 | 3.49 | Moderately soluble | Moderately soluble | Poorly soluble | 3.12 | 3.4 | 0 |
| 18ae | 252.26 | 3 | 0 | 39.44 | 0 | 1.91 | 3.47 | Moderately soluble | Moderately soluble | Poorly soluble | 3.15 | 2.77 | 0 |
| C17 | 293.32 | 4 | 0 | 59.32 | 0 | 1.97 | 3.36 | Soluble | Moderately soluble | Moderately soluble | 3.14 | 2.83 | 0 |
| 18bf | 267.24 | 4 | 0 | 76.03 | 0 | 2.01 | 3.37 | Moderately soluble | Moderately soluble | Moderately soluble | 2.63 | 2.91 | 0 |
| 2f | 344.36 | 4 | 1 | 59.67 | 0 | 2.5 | 4.59 | Moderately soluble | Poorly soluble | Poorly soluble | 4.17 | 3.45 | 0 |
| 18ba | 318.27 | 6 | 0 | 48.67 | 0 | 2.35 | 4.6 | Moderately soluble | Moderately soluble | Poorly soluble | 3.69 | 3.22 | 0 |
| 18bi | 270.71 | 2 | 0 | 30.21 | 0 | 3.04 | 4.42 | Moderately soluble | Moderately soluble | Poorly soluble | 4.02 | 2.93 | 0 |
| C6 | 328.28 | 6 | 0 | 117.94 | 0 | 0.88 | 4.35 | Soluble | Moderately soluble | Moderately soluble | 2.16 | 2.88 | 0 |
| C7 | 358.3 | 7 | 0 | 127.17 | 0 | 0.62 | 4.35 | Moderately soluble | Moderately soluble | Moderately soluble | 2.2 | 3.13 | 0 |
| C15 | 293.32 | 4 | 0 | 59.32 | 0 | 1.97 | 3.36 | Soluble | Moderately soluble | Moderately soluble | 3.11 | 2.86 | 0 |
| 2k | 328.32 | 6 | 3 | 100.13 | 0 | 0.17 | 1.9 | Soluble | Moderately soluble | Moderately soluble | 2.07 | 3.79 | 0 |
| 18bm | 331.16 | 3 | 0 | 39.44 | 0 | 2.54 | 4.23 | Moderately soluble | Moderately soluble | Poorly soluble | 3.86 | 2.84 | 0 |
| 2g | 282.29 | 4 | 0 | 48.67 | 0 | 1.57 | 3.48 | Moderately soluble | Moderately soluble | Poorly soluble | 3.13 | 3.15 | 0 |
| 18bh | 301.13 | 2 | 0 | 30.21 | 0 | 2.91 | 4.22 | Moderately soluble | Moderately soluble | Poorly soluble | 3.87 | 2.87 | 0 |
| 18aq | 330.31 | 6 | 0 | 57.9 | 0 | 1.63 | 4.05 | Moderately soluble | Moderately soluble | Poorly soluble | 3.47 | 3.32 | 0 |
| C4 | 328.28 | 6 | 0 | 117.94 | 0 | 0.88 | 4.35 | Soluble | Moderately soluble | Moderately soluble | 2.13 | 2.94 | 0 |
| C19 | 263.29 | 3 | 0 | 50.09 | 0 | 2.32 | 3.35 | Soluble | Moderately soluble | Moderately soluble | 3.16 | 2.64 | 0 |
| 18av | 327.29 | 6 | 0 | 94.49 | 0 | 0.61 | 3.39 | Moderately soluble | Moderately soluble | Moderately soluble | 2.38 | 3.26 | 0 |
| C9 | 358.3 | 7 | 0 | 127.17 | 0 | 0.62 | 4.35 | Moderately soluble | Moderately soluble | Moderately soluble | 2.11 | 3.15 | 0 |
| C21 | 293.32 | 4 | 0 | 59.32 | 0 | 1.97 | 3.36 | Soluble | Moderately soluble | Moderately soluble | 3.16 | 2.88 | 0 |
| C2 | 313.3 | 5 | 0 | 81.35 | 0 | 1.57 | 3.92 | Soluble | Moderately soluble | Moderately soluble | 2.8 | 2.76 | 0 |
| C1 | 313.3 | 5 | 0 | 81.35 | 0 | 1.57 | 3.92 | Soluble | Moderately soluble | Moderately soluble | 2.76 | 2.77 | 0 |
| 18al | 312.32 | 5 | 0 | 57.9 | 0 | 1.25 | 3.49 | Moderately soluble | Moderately soluble | Poorly soluble | 3.13 | 3.31 | 0 |
| 18aw | 361.19 | 4 | 0 | 48.67 | 0 | 2.19 | 4.24 | Moderately soluble | Moderately soluble | Poorly soluble | 3.76 | 3.18 | 0 |


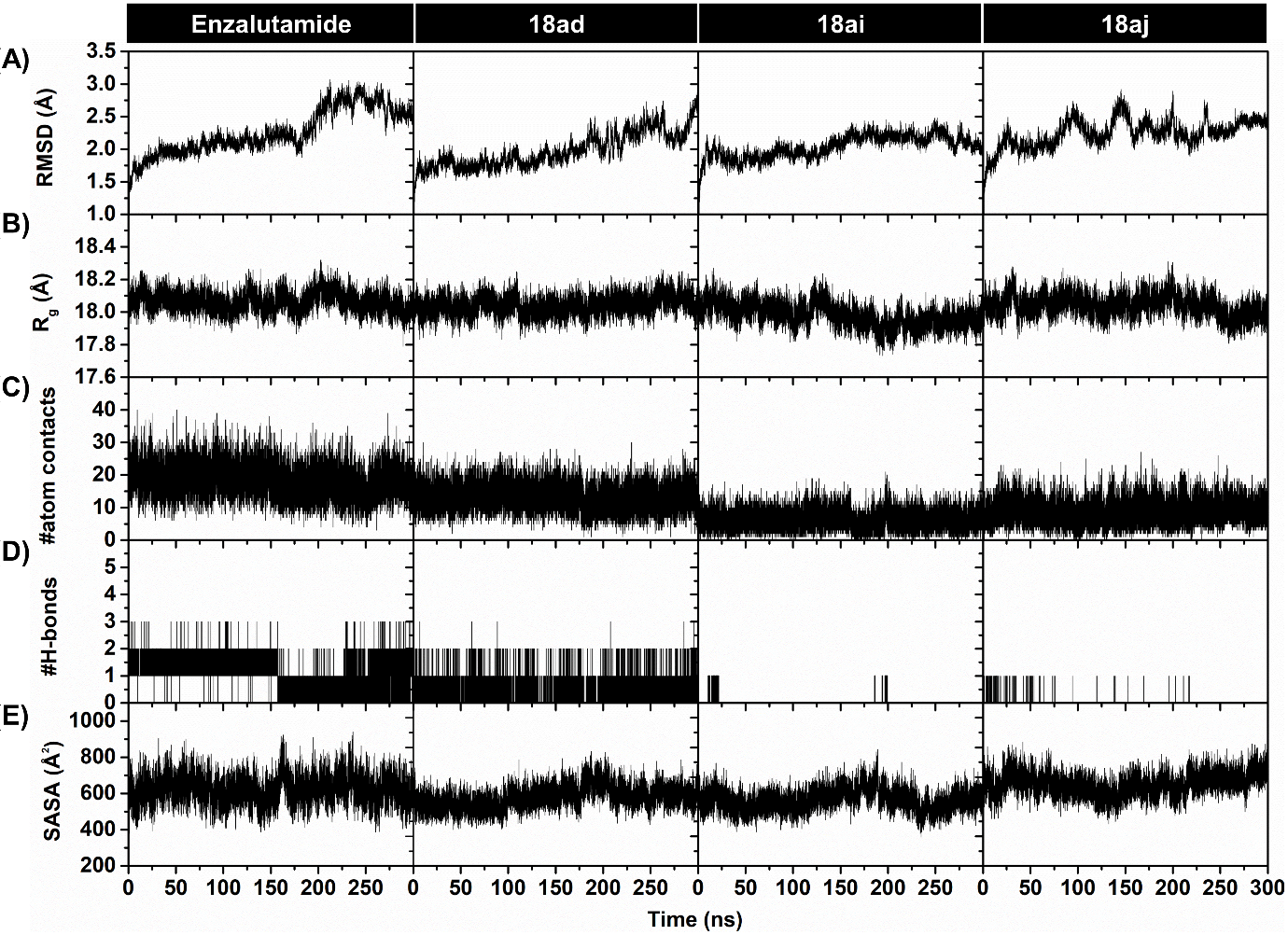


**Figure S2.** Structural stability and interaction profiles of the AR LBD–ligand complexes during 300 ns of MD simulations (rep #2). Time-dependent plots illustrate (**A**) root-mean-square deviation (RMSD), (**B**) radius of gyration (R_g_), (**C**) the number of atomic contacts (#atom contacts), (**D**) the number of intermolecular hydrogen bonds (#H-bonds), and (**E**) solvent-accessible surface area (SASA) for enzalutamide and compounds **18ad**, **18ai**, and **18aj** in complex with the AR LBD.


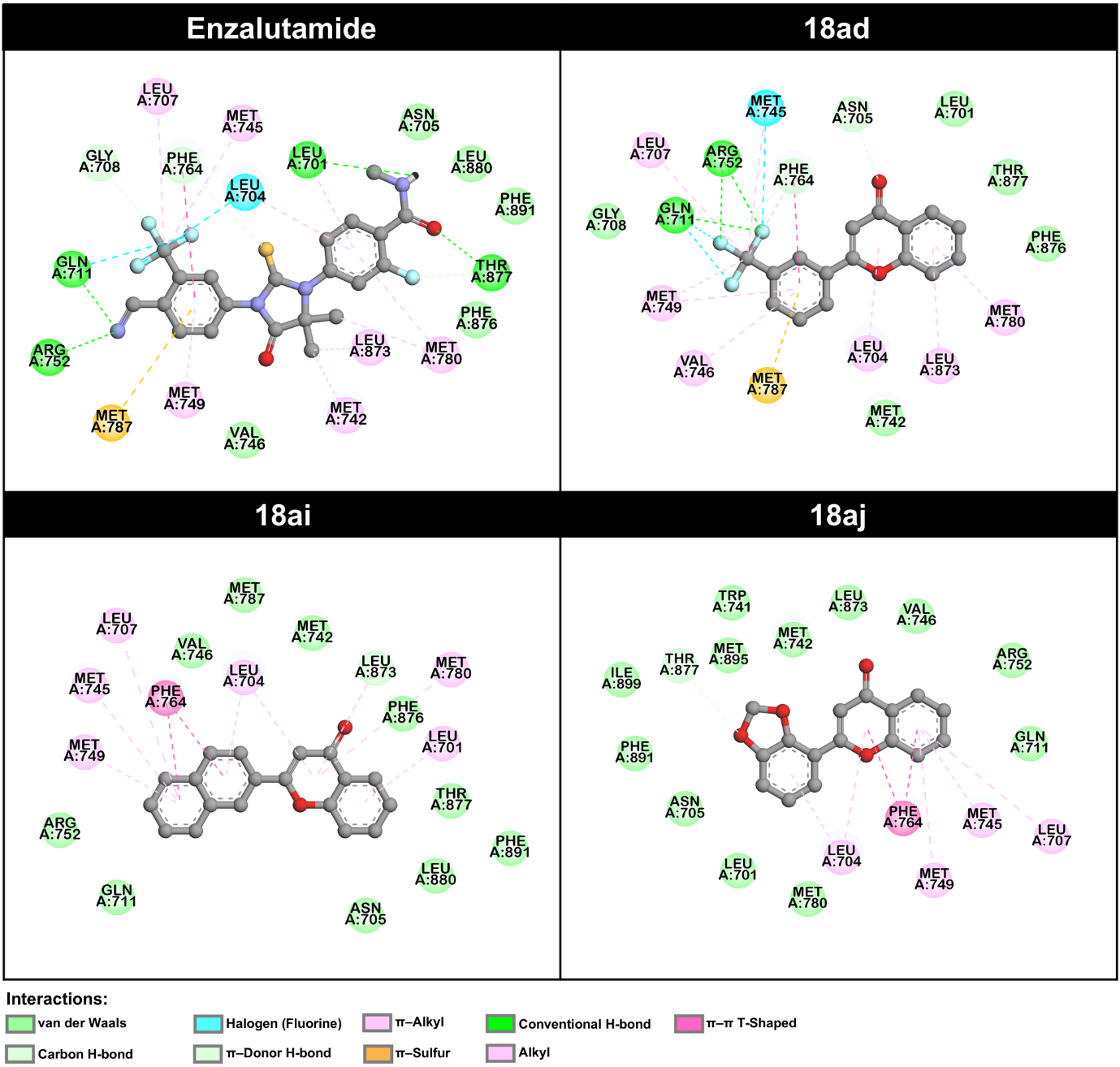


**Figure S3.** 2D interaction diagrams of enzalutamide and compound **18ad**, **18ai**, and **18aj** complexed with the AR mutant H874Y LBD (PDB ID: 2Q7K), obtained from molecular docking.


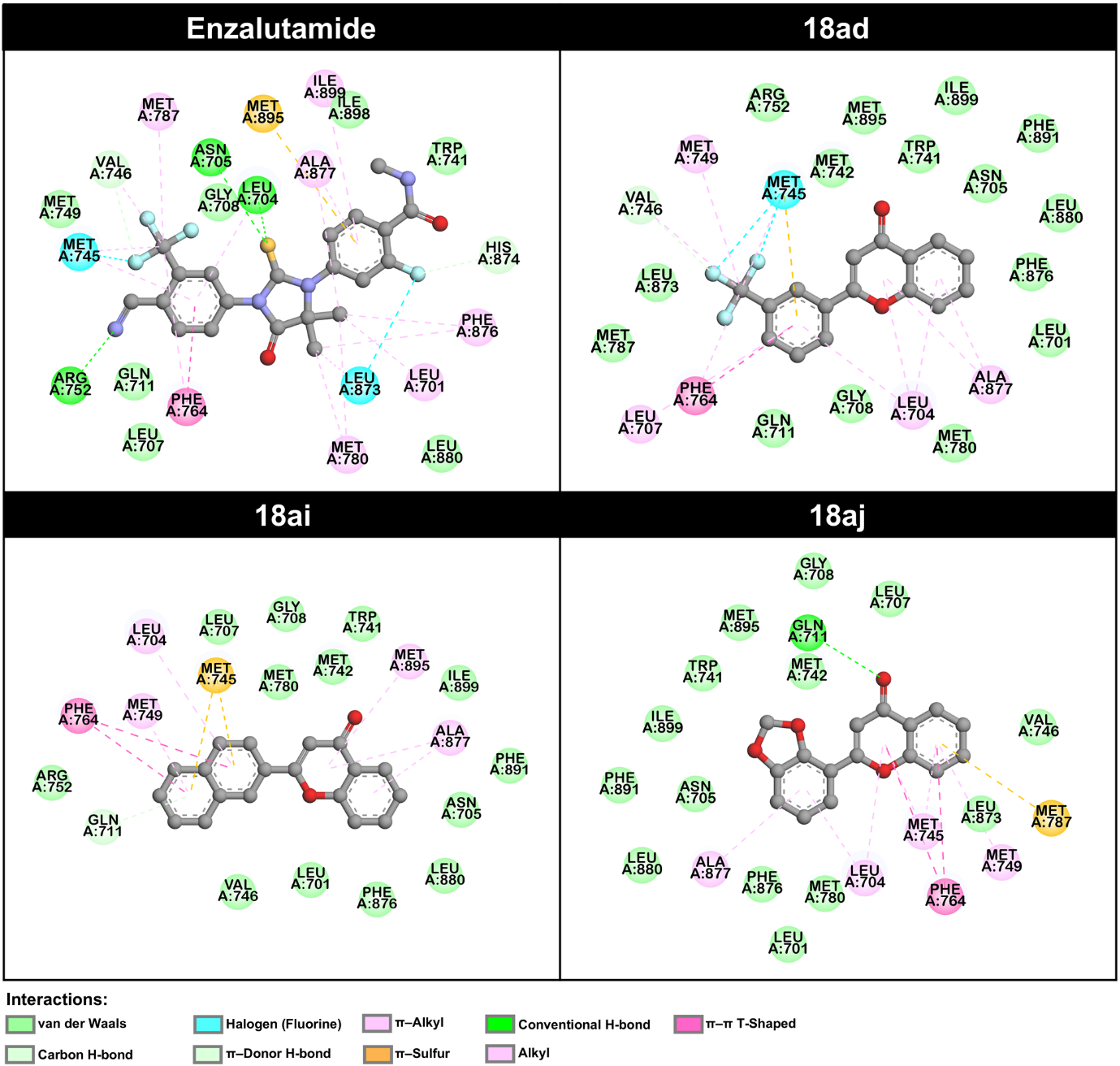


**Figure S4.** 2D interaction diagrams of enzalutamide and compound **18ad**, **18ai**, and **18aj** complexed with the AR mutant T877A LBD (PDB ID: 2AX6), obtained from molecular docking.


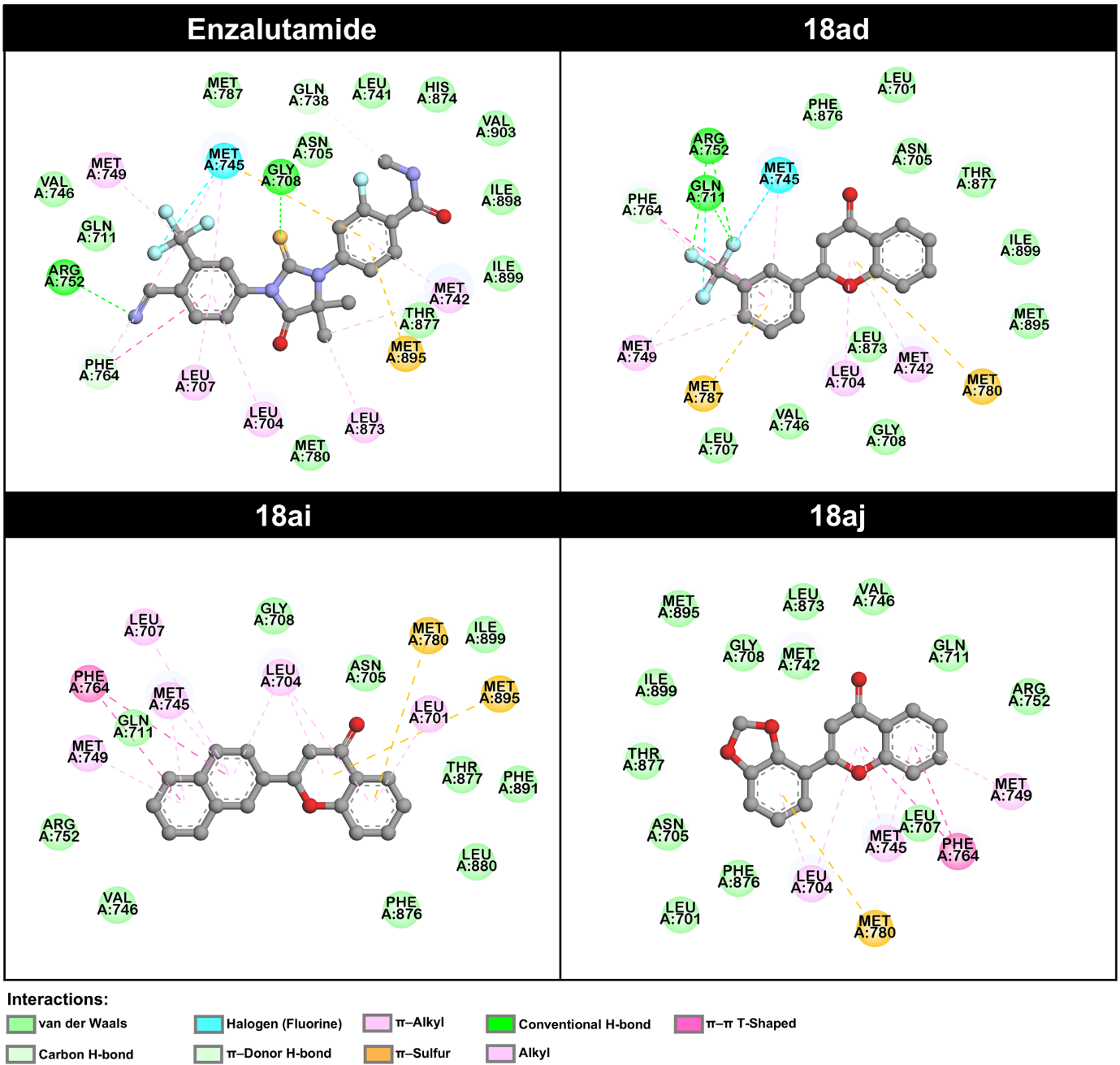


**Figure S5.** 2D interaction diagrams of enzalutamide and compound **18ad**, **18ai**, and **18aj** complexed with the AR mutant W741L LBD (PDB ID: 2AX8), obtained from molecular docking.
